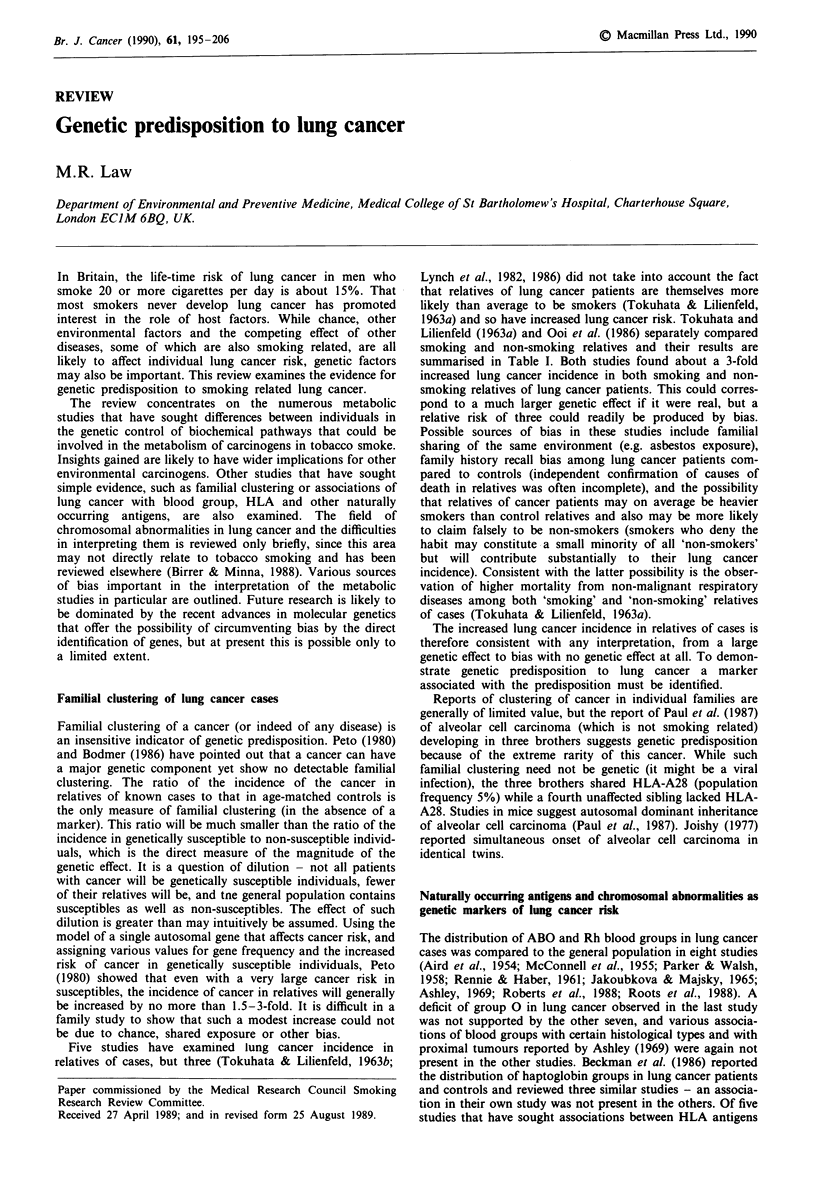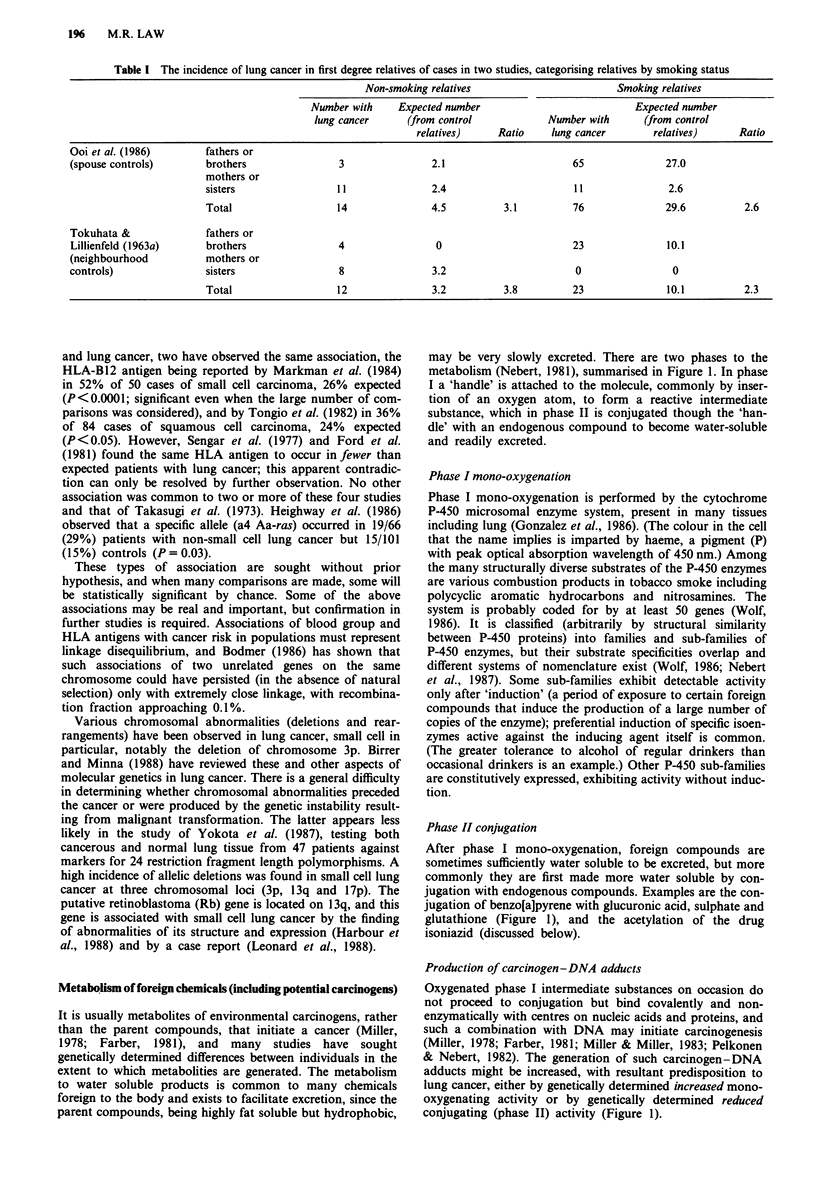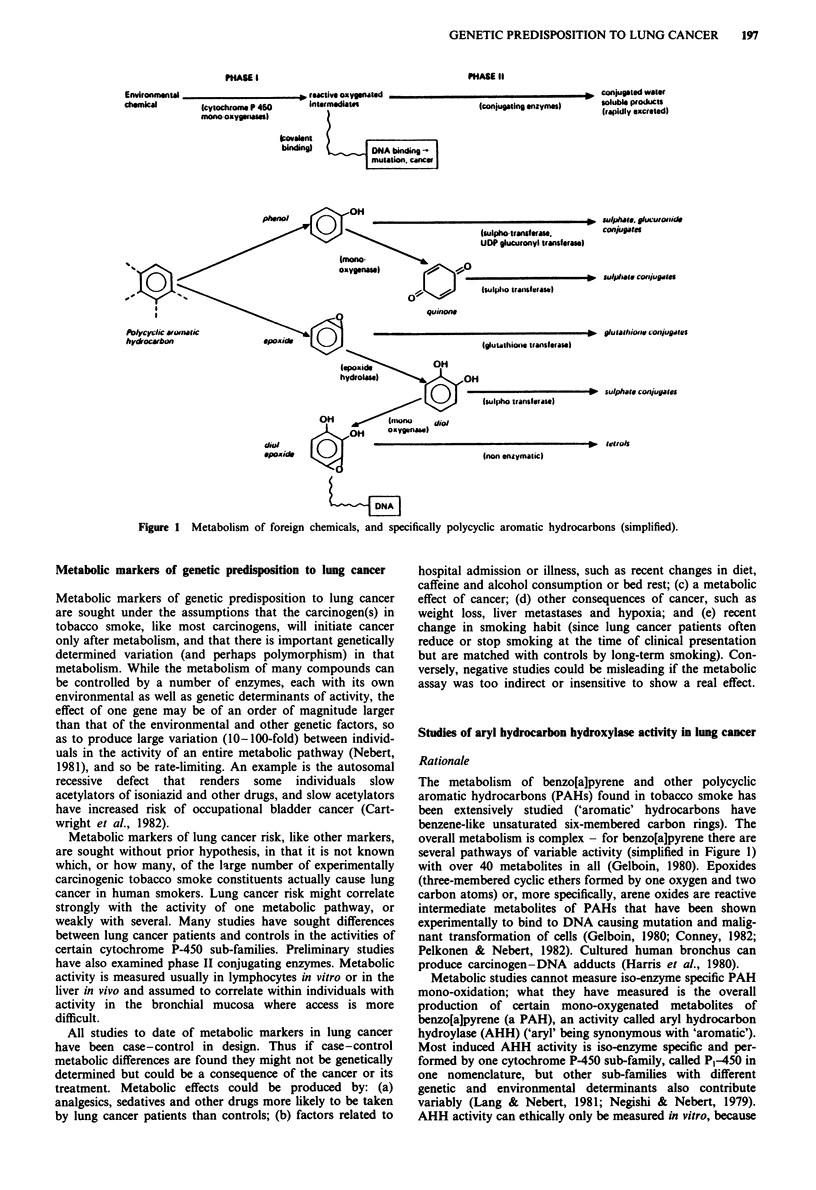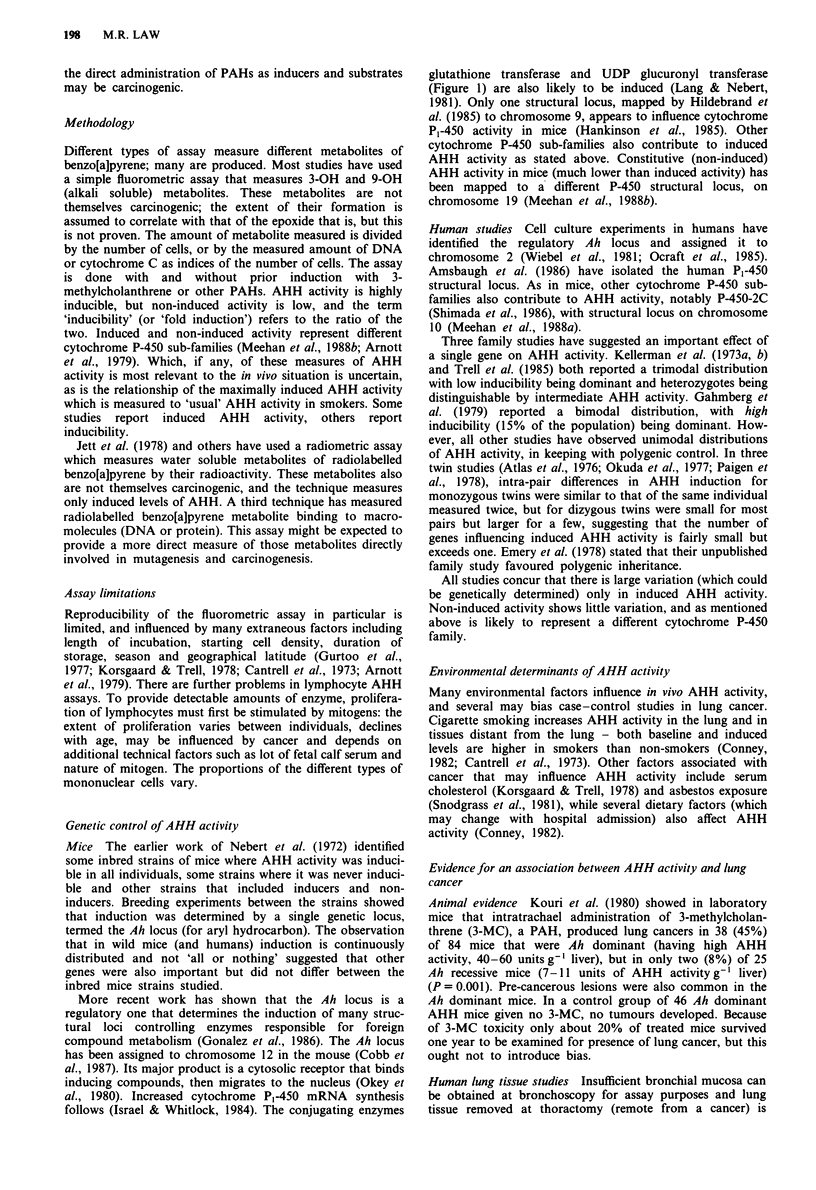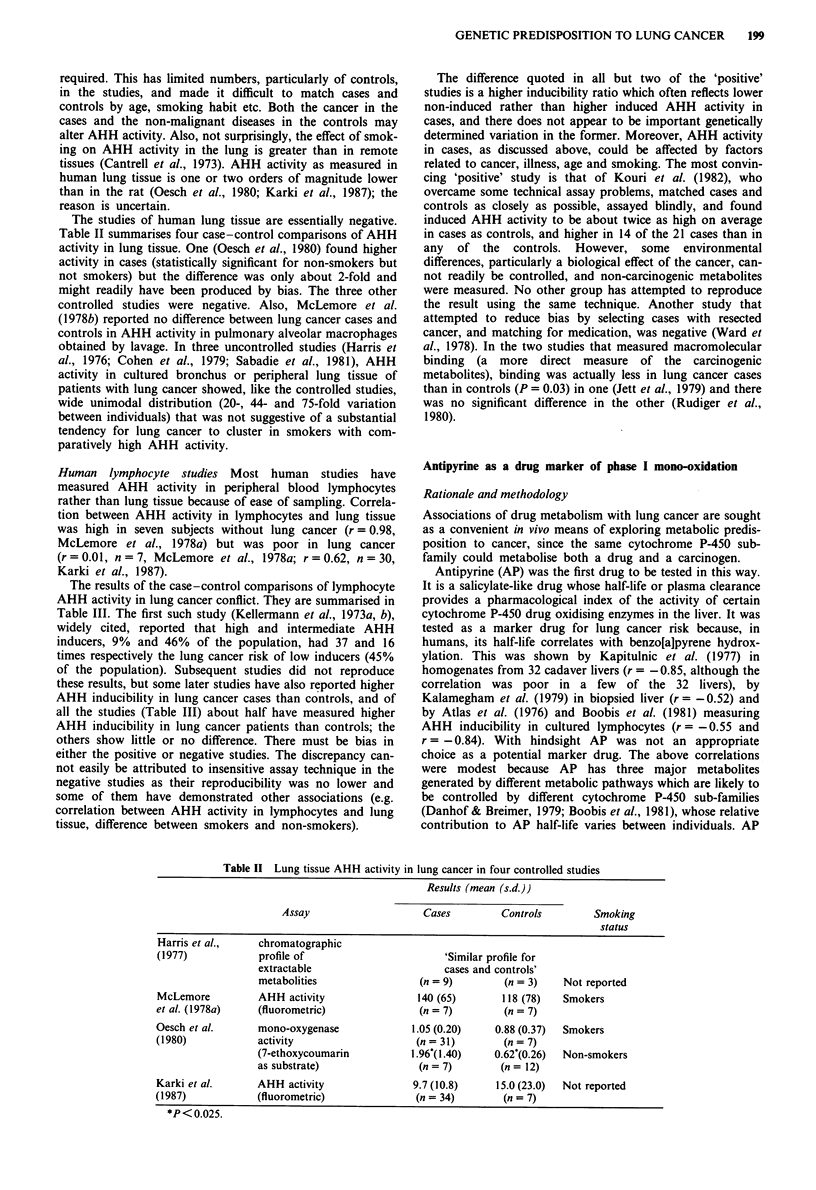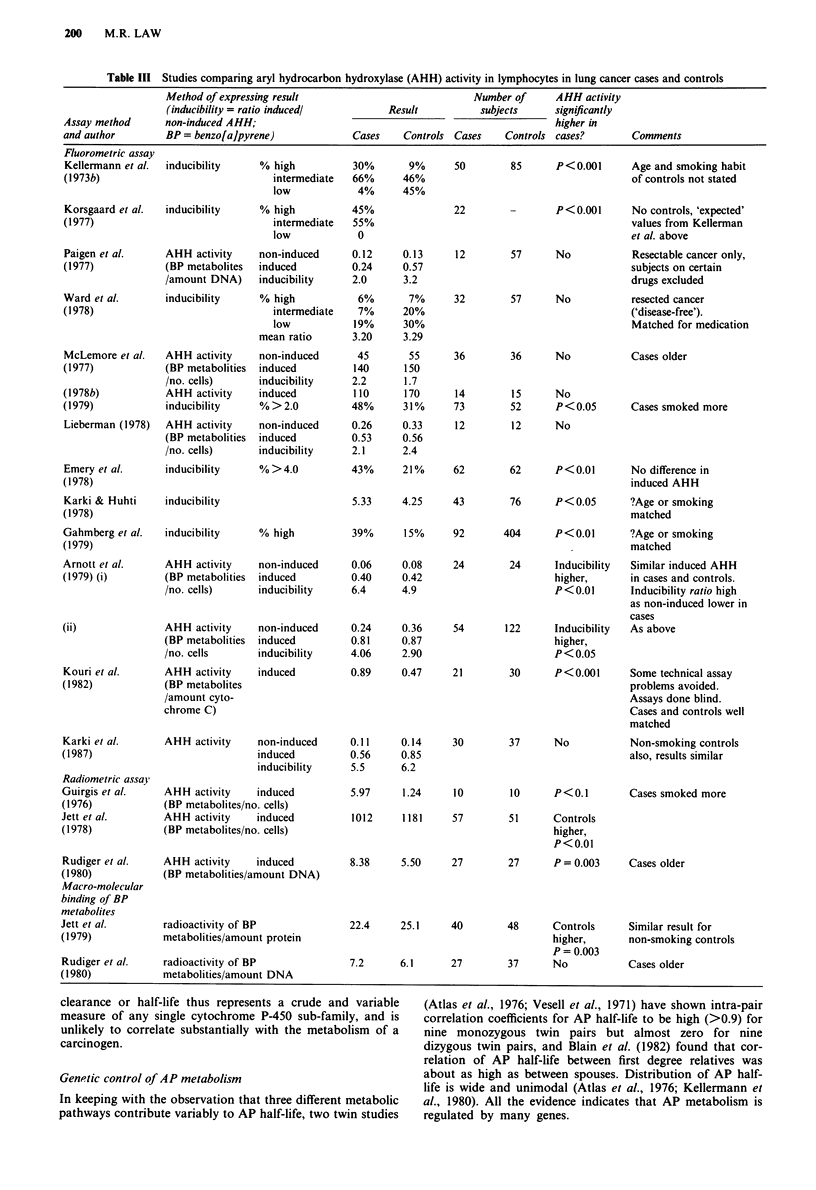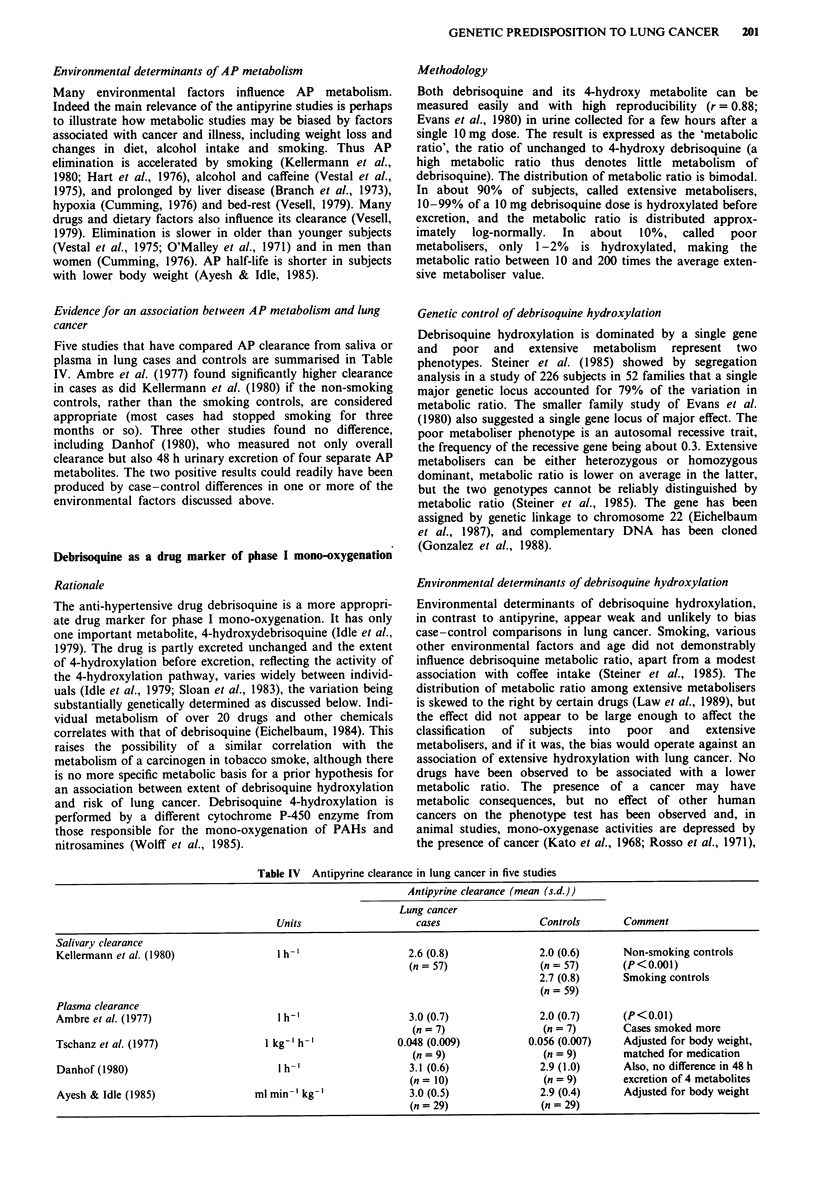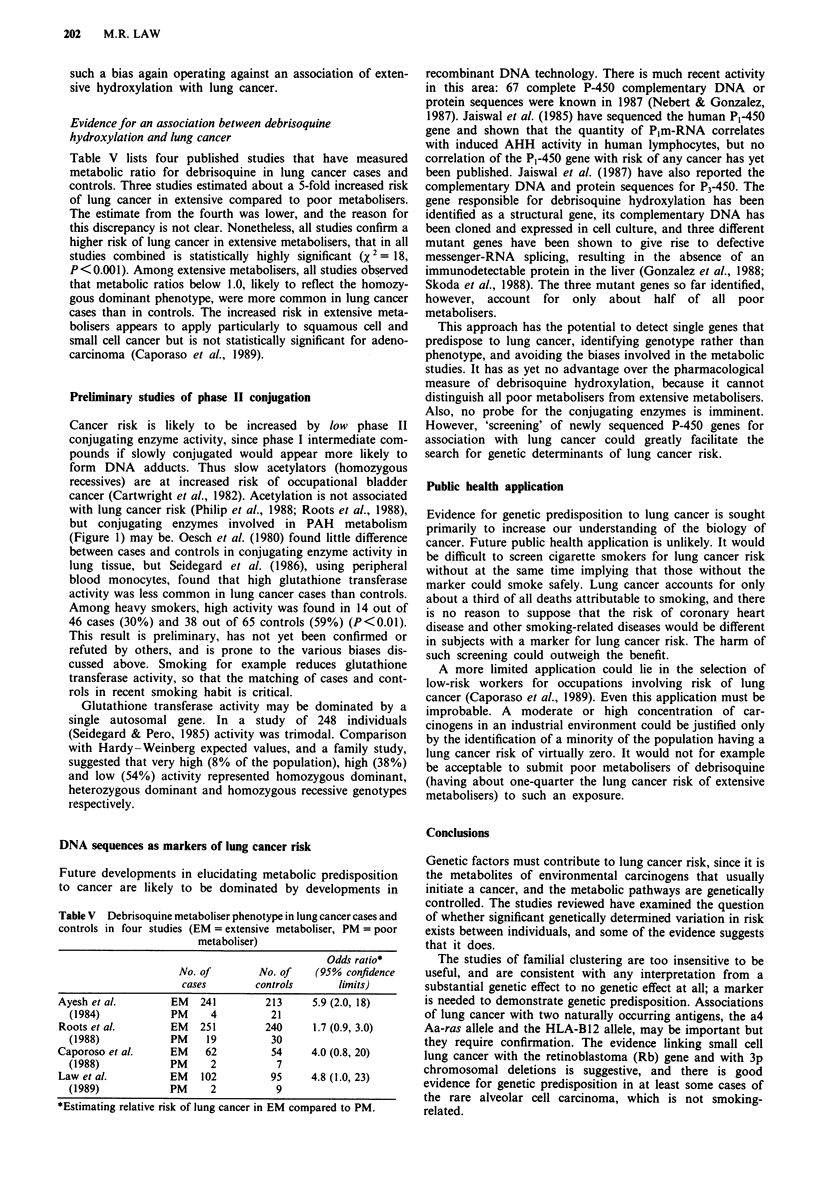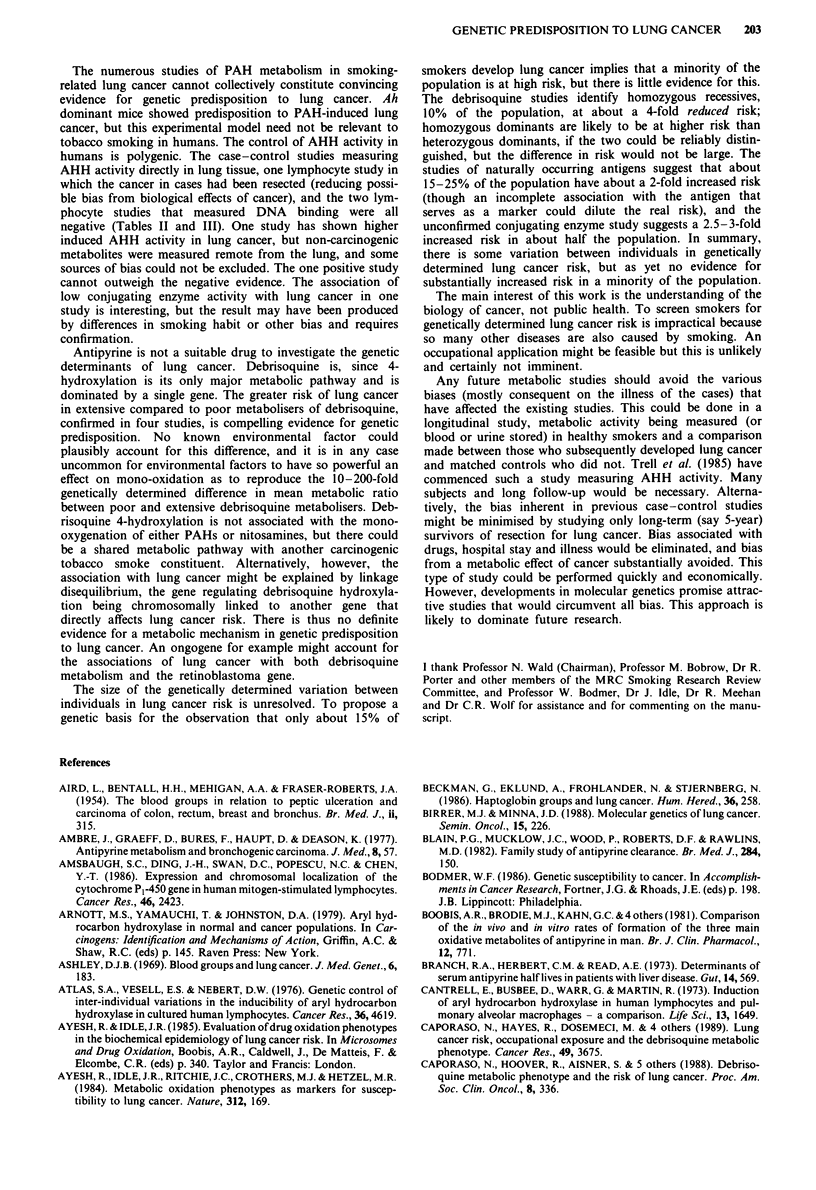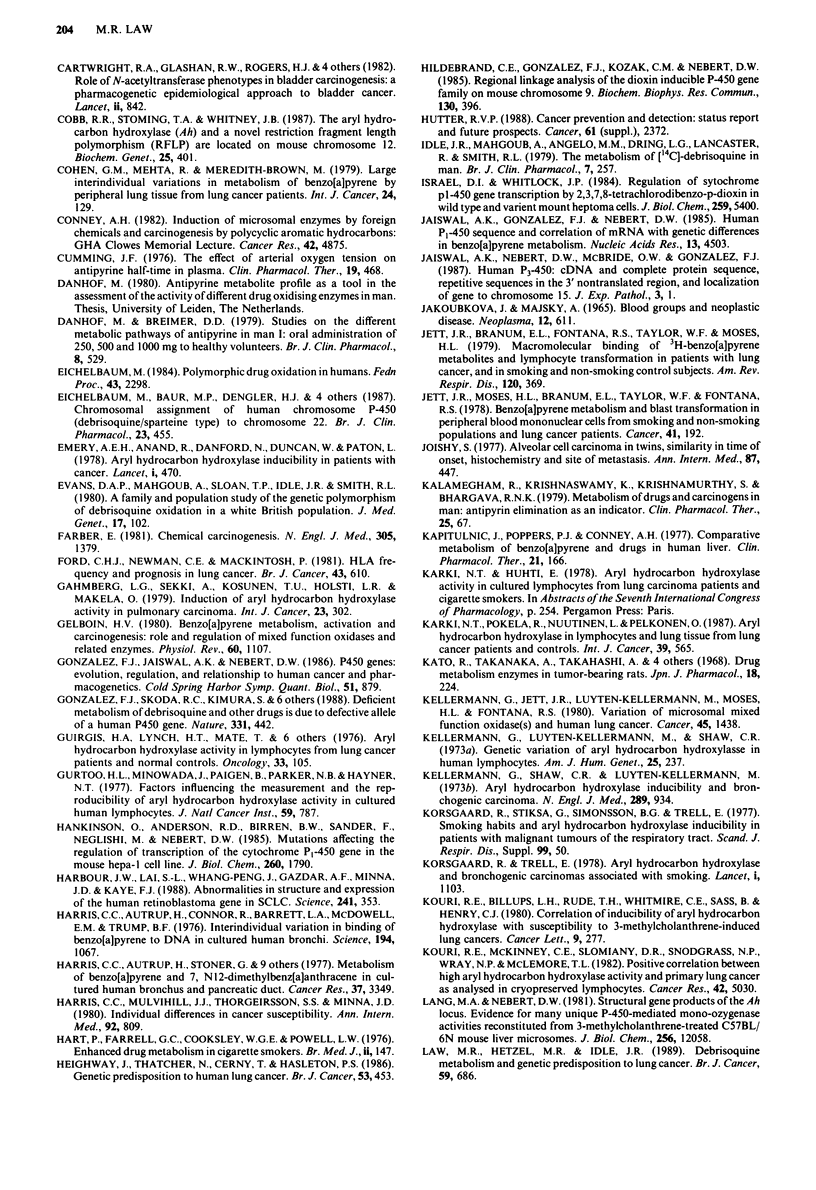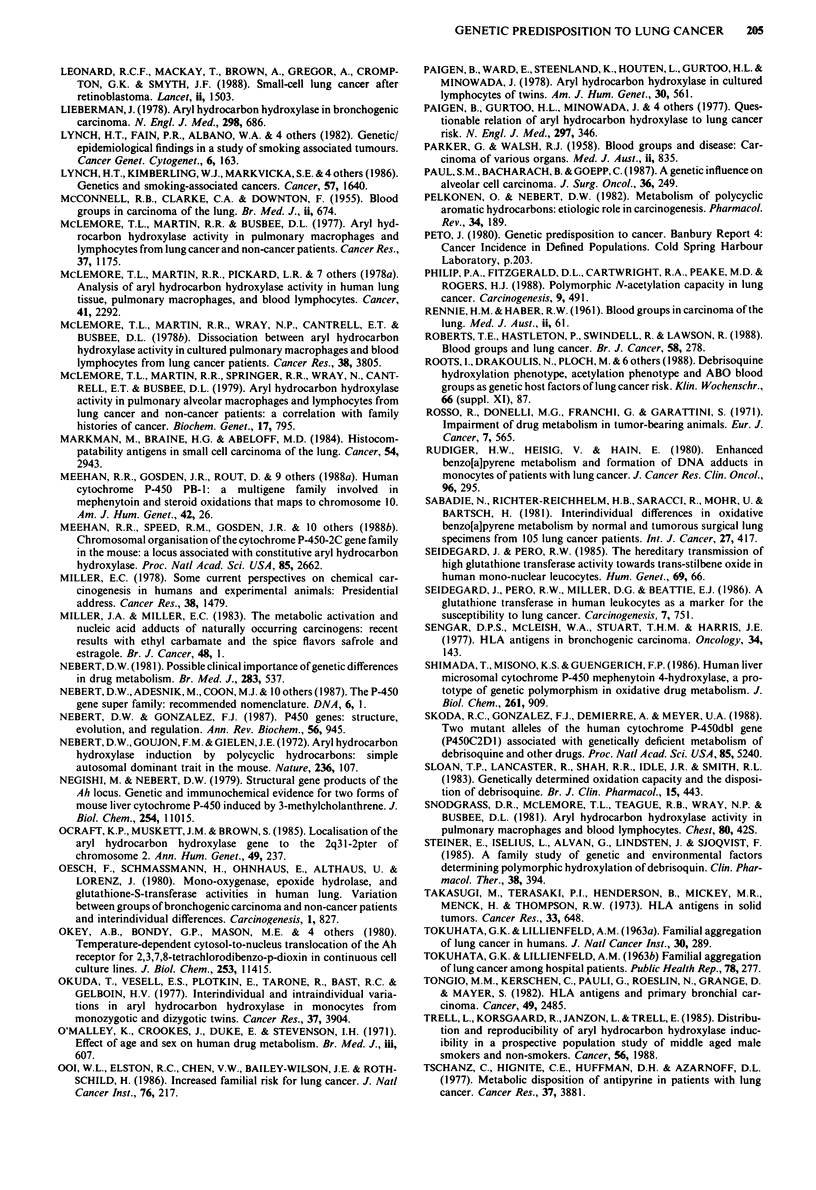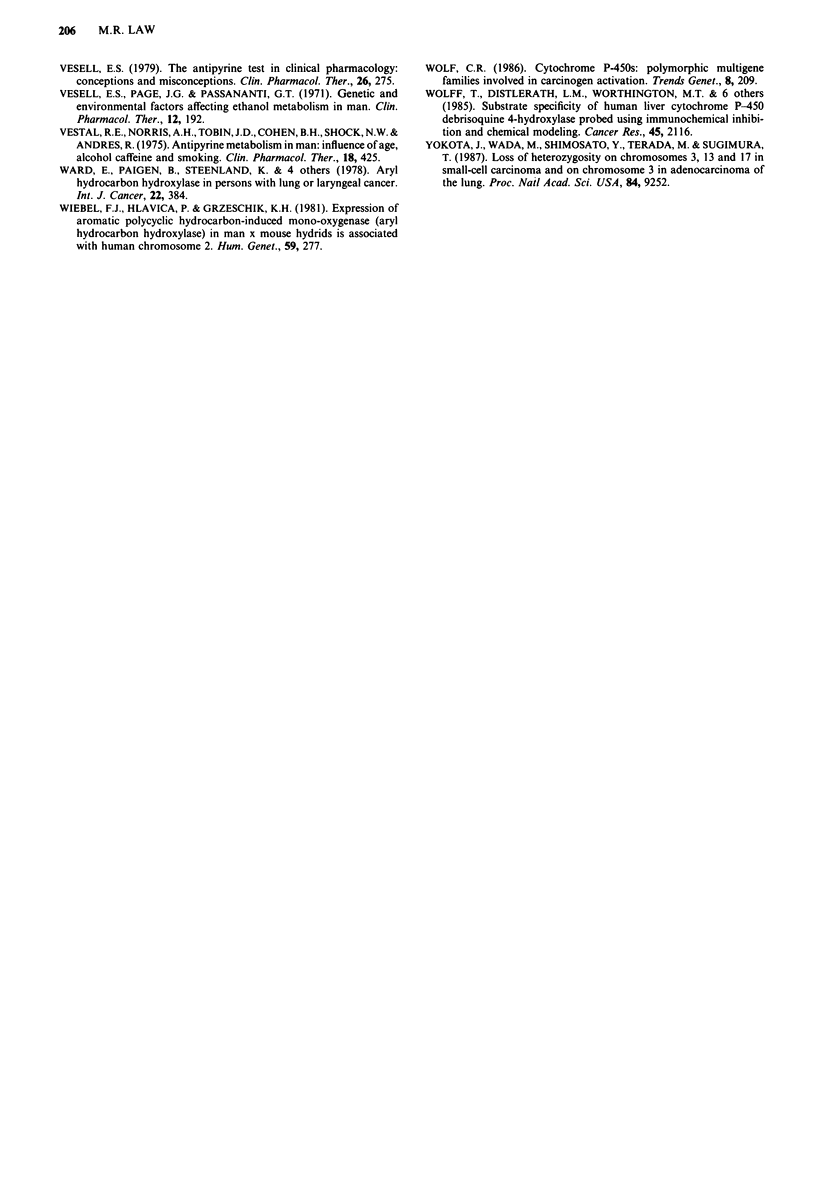# Genetic predisposition to lung cancer.

**DOI:** 10.1038/bjc.1990.37

**Published:** 1990-02

**Authors:** M. R. Law

**Affiliations:** Department of Environmental and Preventive Medicine, Medical College of St Bartholomew's Hospital, London, UK.


Br. J. Cancer (1990), 61, 195-206 © Macmillan Press Ltd., 1990

**REVIEW**

### Genetic predisposition to lung cancer

M.R. Law

*Department of Environmental and Preventive Medicine, Medical College of St Bartholomew's Hospital, Charterhouse Square, London EC1M 6BQ, UK.*

In Britain, the life-time risk of lung cancer in men who smoke 20 or more cigarettes per day is about 15%. That most smokers never develop lung cancer has promoted interest in the role of host factors. While chance, other environmental factors and the competing effect of other diseases, some of which are also smoking related, are all likely to affect individual lung cancer risk, genetic factors may also be important. This review examines the evidence for genetic predisposition to smoking related lung cancer.

The review concentrates on the numerous metabolic studies that have sought differences between individuals in the genetic control of biochemical pathways that could be involved in the metabolism of carcinogens in tobacco smoke. Insights gained are likely to have wider implications for other environmental carcinogens. Other studies that have sought simple evidence, such as familial clustering or associations of lung cancer with blood group, HLA and other naturally occurring antigens, are also examined. The field of chromosomal abnormalities in lung cancer and the difficulties in interpreting them is reviewed only briefly, since this area may not directly relate to tobacco smoking and has been reviewed elsewhere (Birrer & Minna, 1988). Various sources of bias important in the interpretation of the metabolic studies in particular are outlined. Future research is likely to be dominated by the recent advances in molecular genetics that offer the possibility of circumventing bias by the direct identification of genes, but at present this is possible only to a limited extent.

### Familial clustering of lung cancer cases

Familial clustering of a cancer (or indeed of any disease) is an insensitive indicator of genetic predisposition. Peto (1980) and Bodmer (1986) have pointed out that a cancer can have a major genetic component yet show no detectable familial clustering. The ratio of the incidence of the cancer in relatives of known cases to that in age-matched controls is the only measure of familial clustering (in the absence of a marker). This ratio will be much smaller than the ratio of the incidence in genetically susceptible to non-susceptible individuals, which is the direct measure of the magnitude of the genetic effect. It is a question of dilution – not all patients with cancer will be genetically susceptible individuals, fewer of their relatives will be, and tne general population contains susceptibles as well as non-susceptibles. The effect of such dilution is greater than may intuitively be assumed. Using the model of a single autosomal gene that affects cancer risk, and assigning various values for gene frequency and the increased risk of cancer in genetically susceptible individuals, Peto (1980) showed that even with a very large cancer risk in susceptibles, the incidence of cancer in relatives will generally be increased by no more than 1.5–3-fold. It is difficult in a family study to show that such a modest increase could not be due to chance, shared exposure or other bias.

Five studies have examined lung cancer incidence in relatives of cases, but three (Tokuhata & Lilienfeld, 1963*b*; Lynch *et al.*, 1982, 1986) did not take into account the fact that relatives of lung cancer patients are themselves more likely than average to be smokers (Tokuhata & Lilienfeld, 1963*a*) and so have increased lung cancer risk. Tokuhata and Lilienfeld (1963*a*) and Ooi *et al.* (1986) separately compared smoking and non-smoking relatives and their results are summarised in Table I. Both studies found about a 3-fold increased lung cancer incidence in both smoking and non-smoking relatives of lung cancer patients. This could correspond to a much larger genetic effect if it were real, but a relative risk of three could readily be produced by bias. Possible sources of bias in these studies include familial sharing of the same environment (e.g. asbestos exposure), family history recall bias among lung cancer patients compared to controls (independent confirmation of causes of death in relatives was often incomplete), and the possibility that relatives of cancer patients may on average be heavier smokers than control relatives and also may be more likely to claim falsely to be non-smokers (smokers who deny the habit may constitute a small minority of all 'non-smokers' but will contribute substantially to their lung cancer incidence). Consistent with the latter possibility is the observation of higher mortality from non-malignant respiratory diseases among both 'smoking' and 'non-smoking' relatives of cases (Tokuhata & Lilienfeld, 1963*a*).

The increased lung cancer incidence in relatives of cases is therefore consistent with any interpretation, from a large genetic effect to bias with no genetic effect at all. To demonstrate genetic predisposition to lung cancer a marker associated with the predisposition must be identified.

Reports of clustering of cancer in individual families are generally of limited value, but the report of Paul *et al.* (1987) of alveolar cell carcinoma (which is not smoking related) developing in three brothers suggests genetic predisposition because of the extreme rarity of this cancer. While such familial clustering need not be genetic (it might be a viral infection), the three brothers shared HLA-A28 (population frequency 5%) while a fourth unaffected sibling lacked HLA-A28. Studies in mice suggest autosomal dominant inheritance of alveolar cell carcinoma (Paul *et al.*, 1987). Joishy (1977) reported simultaneous onset of alveolar cell carcinoma in identical twins.

### Naturally occurring antigens and chromosomal abnormalities as genetic markers of lung cancer risk

The distribution of ABO and Rh blood groups in lung cancer cases was compared to the general population in eight studies (Aird *et al.*, 1954; McConnell *et al.*, 1955; Parker & Walsh, 1958; Rennie & Haber, 1961; Jakoubkova & Majsky, 1965; Ashley, 1969; Roberts *et al.*, 1988; Roots *et al.*, 1988). A deficit of group O in lung cancer observed in the last study was not supported by the other seven, and various associations of blood groups with certain histological types and with proximal tumours reported by Ashley (1969) were again not present in the other studies. Beckman *et al.* (1986) reported the distribution of haptoglobin groups in lung cancer patients and controls and reviewed three similar studies – an association in their own study was not present in the others. Of five studies that have sought associations between HLA antigens

Paper commissioned by the Medical Research Council Smoking Research Review Committee.
Received 27 April 1989; and in revised form 25 August 1989.

**Table I** The incidence of lung cancer in first degree relatives of cases in two studies, categorising relatives by smoking status

| | | Non-smoking relatives | | | Smoking relatives | | |
|---|---|---|---|---|---|---|---|
| | | Number with lung cancer | Expected number (from control relatives) | Ratio | Number with lung cancer | Expected number (from control relatives) | Ratio |
| Ooi *et al.* (1986) (spouse controls) | fathers or brothers | 3 | 2.1 | | 65 | 27.0 | |
| | mothers or sisters | 11 | 2.4 | | 11 | 2.6 | |
| | Total | 14 | 4.5 | 3.1 | 76 | 29.6 | 2.6 |
| Tokuhata & Lillienfeld (1963*a*) (neighbourhood controls) | fathers or brothers | 4 | 0 | | 23 | 10.1 | |
| | mothers or sisters | 8 | 3.2 | | 0 | 0 | |
| | Total | 12 | 3.2 | 3.8 | 23 | 10.1 | 2.3 |

and lung cancer, two have observed the same association, the HLA-B12 antigen being reported by Markman *et al.* (1984) in 52% of 50 cases of small cell carcinoma, 26% expected ($P < 0.0001$; significant even when the large number of comparisons was considered), and by Tongio *et al.* (1982) in 36% of 84 cases of squamous cell carcinoma, 24% expected ($P < 0.05$). However, Sengar *et al.* (1977) and Ford *et al.* (1981) found the same HLA antigen to occur in *fewer* than expected patients with lung cancer; this apparent contradiction can only be resolved by further observation. No other association was common to two or more of these four studies and that of Takasugi *et al.* (1973). Heighway *et al.* (1986) observed that a specific allele (a4 Aa-*ras*) occurred in 19/66 (29%) patients with non-small cell lung cancer but 15/101 (15%) controls ($P = 0.03$).

These types of association are sought without prior hypothesis, and when many comparisons are made, some will be statistically significant by chance. Some of the above associations may be real and important, but confirmation in further studies is required. Associations of blood group and HLA antigens with cancer risk in populations must represent linkage disequilibrium, and Bodmer (1986) has shown that such associations of two unrelated genes on the same chromosome could have persisted (in the absence of natural selection) only with extremely close linkage, with recombination fraction approaching 0.1%.

Various chromosomal abnormalities (deletions and rearrangements) have been observed in lung cancer, small cell in particular, notably the deletion of chromosome 3p. Birrer and Minna (1988) have reviewed these and other aspects of molecular genetics in lung cancer. There is a general difficulty in determining whether chromosomal abnormalities preceded the cancer or were produced by the genetic instability resulting from malignant transformation. The latter appears less likely in the study of Yokota *et al.* (1987), testing both cancerous and normal lung tissue from 47 patients against markers for 24 restriction fragment length polymorphisms. A high incidence of allelic deletions was found in small cell lung cancer at three chromosomal loci (3p, 13q and 17p). The putative retinoblastoma (Rb) gene is located on 13q, and this gene is associated with small cell lung cancer by the finding of abnormalities of its structure and expression (Harbour *et al.*, 1988) and by a case report (Leonard *et al.*, 1988).

### Metabolism of foreign chemicals (including potential carcinogens)

It is usually metabolites of environmental carcinogens, rather than the parent compounds, that initiate a cancer (Miller, 1978; Farber, 1981), and many studies have sought genetically determined differences between individuals in the extent to which metabolities are generated. The metabolism to water soluble products is common to many chemicals foreign to the body and exists to facilitate excretion, since the parent compounds, being highly fat soluble but hydrophobic, may be very slowly excreted. There are two phases to the metabolism (Nebert, 1981), summarised in Figure 1. In phase I a 'handle' is attached to the molecule, commonly by insertion of an oxygen atom, to form a reactive intermediate substance, which in phase II is conjugated though the 'handle' with an endogenous compound to become water-soluble and readily excreted.

### *Phase I mono-oxygenation*

Phase I mono-oxygenation is performed by the cytochrome P-450 microsomal enzyme system, present in many tissues including lung (Gonzalez *et al.*, 1986). (The colour in the cell that the name implies is imparted by haeme, a pigment (P) with peak optical absorption wavelength of 450 nm.) Among the many structurally diverse substrates of the P-450 enzymes are various combustion products in tobacco smoke including polycyclic aromatic hydrocarbons and nitrosamines. The system is probably coded for by at least 50 genes (Wolf, 1986). It is classified (arbitrarily by structural similarity between P-450 proteins) into families and sub-families of P-450 enzymes, but their substrate specificities overlap and different systems of nomenclature exist (Wolf, 1986; Nebert *et al.*, 1987). Some sub-families exhibit detectable activity only after 'induction' (a period of exposure to certain foreign compounds that induce the production of a large number of copies of the enzyme); preferential induction of specific isoenzymes active against the inducing agent itself is common. (The greater tolerance to alcohol of regular drinkers than occasional drinkers is an example.) Other P-450 sub-families are constitutively expressed, exhibiting activity without induction.

### *Phase II conjugation*

After phase I mono-oxygenation, foreign compounds are sometimes sufficiently water soluble to be excreted, but more commonly they are first made more water soluble by conjugation with endogenous compounds. Examples are the conjugation of benzo[a]pyrene with glucuronic acid, sulphate and glutathione (Figure 1), and the acetylation of the drug isoniazid (discussed below).

### *Production of carcinogen–DNA adducts*

Oxygenated phase I intermediate substances on occasion do not proceed to conjugation but bind covalently and non-enzymatically with centres on nucleic acids and proteins, and such a combination with DNA may initiate carcinogenesis (Miller, 1978; Farber, 1981; Miller & Miller, 1983; Pelkonen & Nebert, 1982). The generation of such carcinogen–DNA adducts might be increased, with resultant predisposition to lung cancer, either by genetically determined *increased* mono-oxygenating activity or by genetically determined *reduced* conjugating (phase II) activity (Figure 1).

Figure 1 Metabolism of foreign chemicals, and specifically polycyclic aromatic hydrocarbons (simplified).

### Metabolic markers of genetic predisposition to lung cancer

Metabolic markers of genetic predisposition to lung cancer are sought under the assumptions that the carcinogen(s) in tobacco smoke, like most carcinogens, will initiate cancer only after metabolism, and that there is important genetically determined variation (and perhaps polymorphism) in that metabolism. While the metabolism of many compounds can be controlled by a number of enzymes, each with its own environmental as well as genetic determinants of activity, the effect of one gene may be of an order of magnitude larger than that of the environmental and other genetic factors, so as to produce large variation (10–100-fold) between individuals in the activity of an entire metabolic pathway (Nebert, 1981), and so be rate-limiting. An example is the autosomal recessive defect that renders some individuals slow acetylators of isoniazid and other drugs, and slow acetylators have increased risk of occupational bladder cancer (Cartwright *et al.*, 1982).

Metabolic markers of lung cancer risk, like other markers, are sought without prior hypothesis, in that it is not known which, or how many, of the large number of experimentally carcinogenic tobacco smoke constituents actually cause lung cancer in human smokers. Lung cancer risk might correlate strongly with the activity of one metabolic pathway, or weakly with several. Many studies have sought differences between lung cancer patients and controls in the activities of certain cytochrome P-450 sub-families. Preliminary studies have also examined phase II conjugating enzymes. Metabolic activity is measured usually in lymphocytes *in vitro* or in the liver *in vivo* and assumed to correlate within individuals with activity in the bronchial mucosa where access is more difficult.

All studies to date of metabolic markers in lung cancer have been case–control in design. Thus if case–control metabolic differences are found they might not be genetically determined but could be a consequence of the cancer or its treatment. Metabolic effects could be produced by: (a) analgesics, sedatives and other drugs more likely to be taken by lung cancer patients than controls; (b) factors related to hospital admission or illness, such as recent changes in diet, caffeine and alcohol consumption or bed rest; (c) a metabolic effect of cancer; (d) other consequences of cancer, such as weight loss, liver metastases and hypoxia; and (e) recent change in smoking habit (since lung cancer patients often reduce or stop smoking at the time of clinical presentation but are matched with controls by long-term smoking). Conversely, negative studies could be misleading if the metabolic assay was too indirect or insensitive to show a real effect.

### Studies of aryl hydrocarbon hydroxylase activity in lung cancer

### *Rationale*

The metabolism of benzo[a]pyrene and other polycyclic aromatic hydrocarbons (PAHs) found in tobacco smoke has been extensively studied ('aromatic' hydrocarbons have benzene-like unsaturated six-membered carbon rings). The overall metabolism is complex – for benzo[a]pyrene there are several pathways of variable activity (simplified in Figure 1) with over 40 metabolites in all (Gelboin, 1980). Epoxides (three-membered cyclic ethers formed by one oxygen and two carbon atoms) or, more specifically, arene oxides are reactive intermediate metabolites of PAHs that have been shown experimentally to bind to DNA causing mutation and malignant transformation of cells (Gelboin, 1980; Conney, 1982; Pelkonen & Nebert, 1982). Cultured human bronchus can produce carcinogen–DNA adducts (Harris *et al.*, 1980).

Metabolic studies cannot measure iso-enzyme specific PAH mono-oxidation; what they have measured is the overall production of certain mono-oxygenated metabolites of benzo[a]pyrene (a PAH), an activity called aryl hydrocarbon hydroylase (AHH) ('aryl' being synonymous with 'aromatic'). Most induced AHH activity is iso-enzyme specific and performed by one cytochrome P-450 sub-family, called $P_1$-450 in one nomenclature, but other sub-families with different genetic and environmental determinants also contribute variably (Lang & Nebert, 1981; Negishi & Nebert, 1979). AHH activity can ethically only be measured *in vitro*, because the direct administration of PAHs as inducers and substrates may be carcinogenic.

*Methodology*

Different types of assay measure different metabolites of benzo[a]pyrene; many are produced. Most studies have used a simple fluorometric assay that measures 3-OH and 9-OH (alkali soluble) metabolites. These metabolites are not themselves carcinogenic; the extent of their formation is assumed to correlate with that of the epoxide that is, but this is not proven. The amount of metabolite measured is divided by the number of cells, or by the measured amount of DNA or cytochrome C as indices of the number of cells. The assay is done with and without prior induction with 3-methylcholanthrene or other PAHs. AHH activity is highly inducible, but non-induced activity is low, and the term 'inducibility' (or 'fold induction') refers to the ratio of the two. Induced and non-induced activity represent different cytochrome P-450 sub-families (Meehan *et al.*, 1988*b*; Arnott *et al.*, 1979). Which, if any, of these measures of AHH activity is most relevant to the *in vivo* situation is uncertain, as is the relationship of the maximally induced AHH activity which is measured to 'usual' AHH activity in smokers. Some studies report induced AHH activity, others report inducibility.

Jett *et al.* (1978) and others have used a radiometric assay which measures water soluble metabolites of radiolabelled benzo[a]pyrene by their radioactivity. These metabolites also are not themselves carcinogenic, and the technique measures only induced levels of AHH. A third technique has measured radiolabelled benzo[a]pyrene metabolite binding to macromolecules (DNA or protein). This assay might be expected to provide a more direct measure of those metabolites directly involved in mutagenesis and carcinogenesis.

*Assay limitations*

Reproducibility of the fluorometric assay in particular is limited, and influenced by many extraneous factors including length of incubation, starting cell density, duration of storage, season and geographical latitude (Gurtoo *et al.*, 1977; Korsgaard & Trell, 1978; Cantrell *et al.*, 1973; Arnott *et al.*, 1979). There are further problems in lymphocyte AHH assays. To provide detectable amounts of enzyme, proliferation of lymphocytes must first be stimulated by mitogens: the extent of proliferation varies between individuals, declines with age, may be influenced by cancer and depends on additional technical factors such as lot of fetal calf serum and nature of mitogen. The proportions of the different types of mononuclear cells vary.

*Genetic control of AHH activity*

*Mice* The earlier work of Nebert *et al.* (1972) identified some inbred strains of mice where AHH activity was inducible in all individuals, some strains where it was never inducible and other strains that included inducers and non-inducers. Breeding experiments between the strains showed that induction was determined by a single genetic locus, termed the *Ah* locus (for aryl hydrocarbon). The observation that in wild mice (and humans) induction is continuously distributed and not 'all or nothing' suggested that other genes were also important but did not differ between the inbred mice strains studied.

More recent work has shown that the *Ah* locus is a regulatory one that determines the induction of many structural loci controlling enzymes responsible for foreign compound metabolism (Gonalez *et al.*, 1986). The *Ah* locus has been assigned to chromosome 12 in the mouse (Cobb *et al.*, 1987). Its major product is a cytosolic receptor that binds inducing compounds, then migrates to the nucleus (Okey *et al.*, 1980). Increased cytochrome $P_1$-450 mRNA synthesis follows (Israel & Whitlock, 1984). The conjugating enzymes glutathione transferase and UDP glucuronyl transferase (Figure 1) are also likely to be induced (Lang & Nebert, 1981). Only one structural locus, mapped by Hildebrand *et al.* (1985) to chromosome 9, appears to influence cytochrome $P_1$-450 activity in mice (Hankinson *et al.*, 1985). Other cytochrome P-450 sub-families also contribute to induced AHH activity as stated above. Constitutive (non-induced) AHH activity in mice (much lower than induced activity) has been mapped to a different P-450 structural locus, on chromosome 19 (Meehan *et al.*, 1988*b*).

*Human studies* Cell culture experiments in humans have identified the regulatory *Ah* locus and assigned it to chromosome 2 (Wiebel *et al.*, 1981; Ocraft *et al.*, 1985). Amsbaugh *et al.* (1986) have isolated the human $P_1$-450 structural locus. As in mice, other cytochrome P-450 sub-families also contribute to AHH activity, notably P-450-2C (Shimada *et al.*, 1986), with structural locus on chromosome 10 (Meehan *et al.*, 1988*a*).

Three family studies have suggested an important effect of a single gene on AHH activity. Kellerman *et al.* (1973*a*, *b*) and Trell *et al.* (1985) both reported a trimodal distribution with low inducibility being dominant and heterozygotes being distinguishable by intermediate AHH activity. Gahmberg *et al.* (1979) reported a bimodal distribution, with *high* inducibility (15% of the population) being dominant. However, all other studies have observed unimodal distributions of AHH activity, in keeping with polygenic control. In three twin studies (Atlas *et al.*, 1976; Okuda *et al.*, 1977; Paigen *et al.*, 1978), intra-pair differences in AHH induction for monozygous twins were similar to that of the same individual measured twice, but for dizygous twins were small for most pairs but larger for a few, suggesting that the number of genes influencing induced AHH activity is fairly small but exceeds one. Emery *et al.* (1978) stated that their unpublished family study favoured polygenic inheritance.

All studies concur that there is large variation (which could be genetically determined) only in induced AHH activity. Non-induced activity shows little variation, and as mentioned above is likely to represent a different cytochrome P-450 family.

*Environmental determinants of AHH activity*

Many environmental factors influence *in vivo* AHH activity, and several may bias case–control studies in lung cancer. Cigarette smoking increases AHH activity in the lung and in tissues distant from the lung – both baseline and induced levels are higher in smokers than non-smokers (Conney, 1982; Cantrell *et al.*, 1973). Other factors associated with cancer that may influence AHH activity include serum cholesterol (Korsgaard & Trell, 1978) and asbestos exposure (Snodgrass *et al.*, 1981), while several dietary factors (which may change with hospital admission) also affect AHH activity (Conney, 1982).

*Evidence for an association between AHH activity and lung cancer*

*Animal evidence* Kouri *et al.* (1980) showed in laboratory mice that intratrachael administration of 3-methylcholanthrene (3-MC), a PAH, produced lung cancers in 38 (45%) of 84 mice that were *Ah* dominant (having high AHH activity, 40–60 units $g^{-1}$ liver), but in only two (8%) of 25 *Ah* recessive mice (7–11 units of AHH activity $g^{-1}$ liver) ($P = 0.001$). Pre-cancerous lesions were also common in the *Ah* dominant mice. In a control group of 46 *Ah* dominant AHH mice given no 3-MC, no tumours developed. Because of 3-MC toxicity only about 20% of treated mice survived one year to be examined for presence of lung cancer, but this ought not to introduce bias.

*Human lung tissue studies* Insufficient bronchial mucosa can be obtained at bronchoscopy for assay purposes and lung tissue removed at thoractomy (remote from a cancer) is required. This has limited numbers, particularly of controls, in the studies, and made it difficult to match cases and controls by age, smoking habit etc. Both the cancer in the cases and the non-malignant diseases in the controls may alter AHH activity. Also, not surprisingly, the effect of smoking on AHH activity in the lung is greater than in remote tissues (Cantrell *et al.*, 1973). AHH activity as measured in human lung tissue is one or two orders of magnitude lower than in the rat (Oesch *et al.*, 1980; Karki *et al.*, 1987); the reason is uncertain.

The studies of human lung tissue are essentially negative. Table II summarises four case-control comparisons of AHH activity in lung tissue. One (Oesch *et al.*, 1980) found higher activity in cases (statistically significant for non-smokers but not smokers) but the difference was only about 2-fold and might readily have been produced by bias. The three other controlled studies were negative. Also, McLemore *et al.* (1978*b*) reported no difference between lung cancer cases and controls in AHH activity in pulmonary alveolar macrophages obtained by lavage. In three uncontrolled studies (Harris *et al.*, 1976; Cohen *et al.*, 1979; Sabadie *et al.*, 1981), AHH activity in cultured bronchus or peripheral lung tissue of patients with lung cancer showed, like the controlled studies, wide unimodal distribution (20-, 44- and 75-fold variation between individuals) that was not suggestive of a substantial tendency for lung cancer to cluster in smokers with comparatively high AHH activity.

*Human lymphocyte studies* Most human studies have measured AHH activity in peripheral blood lymphocytes rather than lung tissue because of ease of sampling. Correlation between AHH activity in lymphocytes and lung tissue was high in seven subjects without lung cancer ($r = 0.98$, McLemore *et al.*, 1978*a*) but was poor in lung cancer ($r = 0.01$, $n = 7$, McLemore *et al.*, 1978*a*; $r = 0.62$, $n = 30$, Karki *et al.*, 1987).

The results of the case-control comparisons of lymphocyte AHH activity in lung cancer conflict. They are summarised in Table III. The first such study (Kellermann *et al.*, 1973*a*, *b*), widely cited, reported that high and intermediate AHH inducers, 9% and 46% of the population, had 37 and 16 times respectively the lung cancer risk of low inducers (45% of the population). Subsequent studies did not reproduce these results, but some later studies have also reported higher AHH inducibility in lung cancer cases than controls, and of all the studies (Table III) about half have measured higher AHH inducibility in lung cancer patients than controls; the others show little or no difference. There must be bias in either the positive or negative studies. The discrepancy cannot easily be attributed to insensitive assay technique in the negative studies as their reproducibility was no lower and some of them have demonstrated other associations (e.g. correlation between AHH activity in lymphocytes and lung tissue, difference between smokers and non-smokers).

The difference quoted in all but two of the 'positive' studies is a higher inducibility ratio which often reflects lower non-induced rather than higher induced AHH activity in cases, and there does not appear to be important genetically determined variation in the former. Moreover, AHH activity in cases, as discussed above, could be affected by factors related to cancer, illness, age and smoking. The most convincing 'positive' study is that of Kouri *et al.* (1982), who overcame some technical assay problems, matched cases and controls as closely as possible, assayed blindly, and found induced AHH activity to be about twice as high on average in cases as controls, and higher in 14 of the 21 cases than in any of the controls. However, some environmental differences, particularly a biological effect of the cancer, cannot readily be controlled, and non-carcinogenic metabolites were measured. No other group has attempted to reproduce the result using the same technique. Another study that attempted to reduce bias by selecting cases with resected cancer, and matching for medication, was negative (Ward *et al.*, 1978). In the two studies that measured macromolecular binding (a more direct measure of the carcinogenic metabolites), binding was actually less in lung cancer cases than in controls ($P = 0.03$) in one (Jett *et al.*, 1979) and there was no significant difference in the other (Rudiger *et al.*, 1980).

### Antipyrine as a drug marker of phase I mono-oxidation

### *Rationale and methodology*

Associations of drug metabolism with lung cancer are sought as a convenient *in vivo* means of exploring metabolic predisposition to cancer, since the same cytochrome P-450 subfamily could metabolise both a drug and a carcinogen.

Antipyrine (AP) was the first drug to be tested in this way. It is a salicylate-like drug whose half-life or plasma clearance provides a pharmacological index of the activity of certain cytochrome P-450 drug oxidising enzymes in the liver. It was tested as a marker drug for lung cancer risk because, in humans, its half-life correlates with benzo[a]pyrene hydroxylation. This was shown by Kapitulnic *et al.* (1977) in homogenates from 32 cadaver livers ($r = -0.85$, although the correlation was poor in a few of the 32 livers), by Kalamegham *et al.* (1979) in biopsied liver ($r = -0.52$) and by Atlas *et al.* (1976) and Boobis *et al.* (1981) measuring AHH inducibility in cultured lymphocytes ($r = -0.55$ and $r = -0.84$). With hindsight AP was not an appropriate choice as a potential marker drug. The above correlations were modest because AP has three major metabolites generated by different metabolic pathways which are likely to be controlled by different cytochrome P-450 sub-families (Danhof & Breimer, 1979; Boobis *et al.*, 1981), whose relative contribution to AP half-life varies between individuals. AP

**Table II** Lung tissue AHH activity in lung cancer in four controlled studies

| | Assay | Results (mean (s.d.)) Cases | Controls | Smoking status |
|---|---|---|---|---|
| Harris *et al.*, (1977) | chromatographic profile of extractable metabolities | 'Similar profile for cases and controls' ($n = 9$) | ($n = 3$) | Not reported |
| McLemore *et al.* (1978*a*) | AHH activity (fluorometric) | 140 (65) ($n = 7$) | 118 (78) ($n = 7$) | Smokers |
| Oesch *et al.* (1980) | mono-oxygenase activity (7-ethoxycoumarin as substrate) | 1.05 (0.20) ($n = 31$) | 0.88 (0.37) ($n = 7$) | Smokers |
| | | 1.96*(1.40) ($n = 7$) | 0.62*(0.26) ($n = 12$) | Non-smokers |
| Karki *et al.* (1987) | AHH activity (fluorometric) | 9.7 (10.8) ($n = 34$) | 15.0 (23.0) ($n = 7$) | Not reported |

*$P < 0.025$.

**Table III** Studies comparing aryl hydrocarbon hydroxylase (AHH) activity in lymphocytes in lung cancer cases and controls

| *Assay method and author* | *Method of expressing result (inducibility = ratio induced/non-induced AHH; BP = benzo[a]pyrene)* | | *Result* | | *Number of subjects* | | *AHH activity significantly higher in cases?* | *Comments* |
|---|---|---|---|---|---|---|---|---|
| | | | *Cases* | *Controls* | *Cases* | *Controls* | | |
| *Fluorometric assay* | | | | | | | | |
| Kellermann *et al.* (1973*b*) | inducibility | % high | 30% | 9% | 50 | 85 | $P<0.001$ | Age and smoking habit of controls not stated |
| | | intermediate | 66% | 46% | | | | |
| | | low | 4% | 45% | | | | |
| Korsgaard *et al.* (1977) | inducibility | % high | 45% | | 22 | – | $P<0.001$ | No controls, 'expected' values from Kellerman *et al.* above |
| | | intermediate | 55% | | | | | |
| | | low | 0 | | | | | |
| Paigen *et al.* (1977) | AHH activity (BP metabolites /amount DNA) | non-induced | 0.12 | 0.13 | 12 | 57 | No | Resectable cancer only, subjects on certain drugs excluded |
| | | induced | 0.24 | 0.57 | | | | |
| | | inducibility | 2.0 | 3.2 | | | | |
| Ward *et al.* (1978) | inducibility | % high | 6% | 7% | 32 | 57 | No | resected cancer ('disease-free'). Matched for medication |
| | | intermediate | 7% | 20% | | | | |
| | | low | 19% | 30% | | | | |
| | | mean ratio | 3.20 | 3.29 | | | | |
| McLemore *et al.* (1977) | AHH activity (BP metabolities /no. cells) | non-induced | 45 | 55 | 36 | 36 | No | Cases older |
| | | induced | 140 | 150 | | | | |
| | | inducibility | 2.2 | 1.7 | | | | |
| (1978*b*) | AHH activity | induced | 110 | 170 | 14 | 15 | No | |
| (1979) | inducibility | %>2.0 | 48% | 31% | 73 | 52 | $P<0.05$ | Cases smoked more |
| Lieberman (1978) | AHH activity (BP metabolities /no. cells) | non-induced | 0.26 | 0.33 | 12 | 12 | No | |
| | | induced | 0.53 | 0.56 | | | | |
| | | inducibility | 2.1 | 2.4 | | | | |
| Emery *et al.* (1978) | inducibility | %>4.0 | 43% | 21% | 62 | 62 | $P<0.01$ | No difference in induced AHH |
| Karki & Huhti (1978) | inducibility | | 5.33 | 4.25 | 43 | 76 | $P<0.05$ | ?Age or smoking matched |
| Gahmberg *et al.* (1979) | inducibility | % high | 39% | 15% | 92 | 404 | $P<0.01$ | ?Age or smoking matched |
| Arnott *et al.* (1979) (i) | AHH activity (BP metabolities /no. cells) | non-induced | 0.06 | 0.08 | 24 | 24 | Inducibility higher, $P<0.01$ | Similar induced AHH in cases and controls. Inducibility *ratio* high as non-induced lower in cases |
| | | induced | 0.40 | 0.42 | | | | |
| | | inducibility | 6.4 | 4.9 | | | | |
| (ii) | AHH activity (BP metabolities /no. cells | non-induced | 0.24 | 0.36 | 54 | 122 | Inducibility higher, $P<0.05$ | As above |
| | | induced | 0.81 | 0.87 | | | | |
| | | inducibility | 4.06 | 2.90 | | | | |
| Kouri *et al.* (1982) | AHH activity (BP metabolites /amount cyto-chrome C) | induced | 0.89 | 0.47 | 21 | 30 | $P<0.001$ | Some technical assay problems avoided. Assays done blind. Cases and controls well matched |
| Karki *et al.* (1987) | AHH activity | non-induced | 0.11 | 0.14 | 30 | 37 | No | Non-smoking controls also, results similar |
| | | induced | 0.56 | 0.85 | | | | |
| | | inducibility | 5.5 | 6.2 | | | | |
| *Radiometric assay* | | | | | | | | |
| Guirgis *et al.* (1976) | AHH activity (BP metabolites/no. cells) | induced | 5.97 | 1.24 | 10 | 10 | $P<0.1$ | Cases smoked more |
| Jett *et al.* (1978) | AHH activity (BP metabolites/no. cells) | induced | 1012 | 1181 | 57 | 51 | Controls higher, $P<0.01$ | |
| Rudiger *et al.* (1980) | AHH activity (BP metabolities/amount DNA) | induced | 8.38 | 5.50 | 27 | 27 | $P=0.003$ | Cases older |
| *Macro-molecular binding of BP metabolites* | | | | | | | | |
| Jett *et al.* (1979) | radioactivity of BP metabolities/amount protein | | 22.4 | 25.1 | 40 | 48 | Controls higher, $P=0.003$ | Similar result for non-smoking controls |
| Rudiger *et al.* (1980) | radioactivity of BP metabolities/amount DNA | | 7.2 | 6.1 | 27 | 37 | No | Cases older |

clearance or half-life thus represents a crude and variable measure of any single cytochrome P-450 sub-family, and is unlikely to correlate substantially with the metabolism of a carcinogen.

### *Genetic control of AP metabolism*

In keeping with the observation that three different metabolic pathways contribute variably to AP half-life, two twin studies (Atlas *et al.*, 1976; Vesell *et al.*, 1971) have shown intra-pair correlation coefficients for AP half-life to be high (>0.9) for nine monozygous twin pairs but almost zero for nine dizygous twin pairs, and Blain *et al.* (1982) found that correlation of AP half-life between first degree relatives was about as high as between spouses. Distribution of AP half-life is wide and unimodal (Atlas *et al.*, 1976; Kellermann *et al.*, 1980). All the evidence indicates that AP metabolism is regulated by many genes.

*Environmental determinants of AP metabolism*

Many environmental factors influence AP metabolism. Indeed the main relevance of the antipyrine studies is perhaps to illustrate how metabolic studies may be biased by factors associated with cancer and illness, including weight loss and changes in diet, alcohol intake and smoking. Thus AP elimination is accelerated by smoking (Kellermann *et al.*, 1980; Hart *et al.*, 1976), alcohol and caffeine (Vestal *et al.*, 1975), and prolonged by liver disease (Branch *et al.*, 1973), hypoxia (Cumming, 1976) and bed-rest (Vesell, 1979). Many drugs and dietary factors also influence its clearance (Vesell, 1979). Elimination is slower in older than younger subjects (Vestal *et al.*, 1975; O'Malley *et al.*, 1971) and in men than women (Cumming, 1976). AP half-life is shorter in subjects with lower body weight (Ayesh & Idle, 1985).

*Evidence for an association between AP metabolism and lung cancer*

Five studies that have compared AP clearance from saliva or plasma in lung cases and controls are summarised in Table IV. Ambre *et al.* (1977) found significantly higher clearance in cases as did Kellermann *et al.* (1980) if the non-smoking controls, rather than the smoking controls, are considered appropriate (most cases had stopped smoking for three months or so). Three other studies found no difference, including Danhof (1980), who measured not only overall clearance but also 48 h urinary excretion of four separate AP metabolites. The two positive results could readily have been produced by case–control differences in one or more of the environmental factors discussed above.

**Debrisoquine as a drug marker of phase I mono-oxygenation**

*Rationale*

The anti-hypertensive drug debrisoquine is a more appropriate drug marker for phase I mono-oxygenation. It has only one important metabolite, 4-hydroxydebrisoquine (Idle *et al.*, 1979). The drug is partly excreted unchanged and the extent of 4-hydroxylation before excretion, reflecting the activity of the 4-hydroxylation pathway, varies widely between individuals (Idle *et al.*, 1979; Sloan *et al.*, 1983), the variation being substantially genetically determined as discussed below. Individual metabolism of over 20 drugs and other chemicals correlates with that of debrisoquine (Eichelbaum, 1984). This raises the possibility of a similar correlation with the metabolism of a carcinogen in tobacco smoke, although there is no more specific metabolic basis for a prior hypothesis for an association between extent of debrisoquine hydroxylation and risk of lung cancer. Debrisoquine 4-hydroxylation is performed by a different cytochrome P-450 enzyme from those responsible for the mono-oxygenation of PAHs and nitrosamines (Wolff *et al.*, 1985).

*Methodology*

Both debrisoquine and its 4-hydroxy metabolite can be measured easily and with high reproducibility ($r = 0.88$; Evans *et al.*, 1980) in urine collected for a few hours after a single 10 mg dose. The result is expressed as the 'metabolic ratio', the ratio of unchanged to 4-hydroxy debrisoquine (a high metabolic ratio thus denotes little metabolism of debrisoquine). The distribution of metabolic ratio is bimodal. In about 90% of subjects, called extensive metabolisers, 10–99% of a 10 mg debrisoquine dose is hydroxylated before excretion, and the metabolic ratio is distributed approximately log-normally. In about 10%, called poor metabolisers, only 1–2% is hydroxylated, making the metabolic ratio between 10 and 200 times the average extensive metaboliser value.

*Genetic control of debrisoquine hydroxylation*

Debrisoquine hydroxylation is dominated by a single gene and poor and extensive metabolism represent two phenotypes. Steiner *et al.* (1985) showed by segregation analysis in a study of 226 subjects in 52 families that a single major genetic locus accounted for 79% of the variation in metabolic ratio. The smaller family study of Evans *et al.* (1980) also suggested a single gene locus of major effect. The poor metaboliser phenotype is an autosomal recessive trait, the frequency of the recessive gene being about 0.3. Extensive metabolisers can be either heterozygous or homozygous dominant, metabolic ratio is lower on average in the latter, but the two genotypes cannot be reliably distinguished by metabolic ratio (Steiner *et al.*, 1985). The gene has been assigned by genetic linkage to chromosome 22 (Eichelbaum *et al.*, 1987), and complementary DNA has been cloned (Gonzalez *et al.*, 1988).

*Environmental determinants of debrisoquine hydroxylation*

Environmental determinants of debrisoquine hydroxylation, in contrast to antipyrine, appear weak and unlikely to bias case–control comparisons in lung cancer. Smoking, various other environmental factors and age did not demonstrably influence debrisoquine metabolic ratio, apart from a modest association with coffee intake (Steiner *et al.*, 1985). The distribution of metabolic ratio among extensive metabolisers is skewed to the right by certain drugs (Law *et al.*, 1989), but the effect did not appear to be large enough to affect the classification of subjects into poor and extensive metabolisers, and if it was, the bias would operate against an association of extensive hydroxylation with lung cancer. No drugs have been observed to be associated with a lower metabolic ratio. The presence of a cancer may have metabolic consequences, but no effect of other human cancers on the phenotype test has been observed and, in animal studies, mono-oxygenase activities are depressed by the presence of cancer (Kato *et al.*, 1968; Rosso *et al.*, 1971),

**Table IV** Antipyrine clearance in lung cancer in five studies

| | | Antipyrine clearance (mean (s.d.)) | | |
|---|---|---|---|---|
| | *Units* | *Lung cancer cases* | *Controls* | *Comment* |
| *Salivary clearance* | | | | |
| Kellermann *et al.* (1980) | $l\ h^{-1}$ | 2.6 (0.8) ($n = 57$) | 2.0 (0.6) ($n = 57$) | Non-smoking controls ($P < 0.001$) |
| | | | 2.7 (0.8) ($n = 59$) | Smoking controls |
| *Plasma clearance* | | | | |
| Ambre *et al.* (1977) | $l\ h^{-1}$ | 3.0 (0.7) ($n = 7$) | 2.0 (0.7) ($n = 7$) | ($P < 0.01$) Cases smoked more |
| Tschanz *et al.* (1977) | $l\ kg^{-1}\ h^{-1}$ | 0.048 (0.009) ($n = 9$) | 0.056 (0.007) ($n = 9$) | Adjusted for body weight, matched for medication |
| Danhof (1980) | $l\ h^{-1}$ | 3.1 (0.6) ($n = 10$) | 2.9 (1.0) ($n = 9$) | Also, no difference in 48 h excretion of 4 metabolites |
| Ayesh & Idle (1985) | $ml\ min^{-1}\ kg^{-1}$ | 3.0 (0.5) ($n = 29$) | 2.9 (0.4) ($n = 29$) | Adjusted for body weight |

such a bias again operating against an association of extensive hydroxylation with lung cancer.

*Evidence for an association between debrisoquine hydroxylation and lung cancer*

Table V lists four published studies that have measured metabolic ratio for debrisoquine in lung cancer cases and controls. Three studies estimated about a 5-fold increased risk of lung cancer in extensive compared to poor metabolisers. The estimate from the fourth was lower, and the reason for this discrepancy is not clear. Nonetheless, all studies confirm a higher risk of lung cancer in extensive metabolisers, that in all studies combined is statistically highly significant ($\chi^2 = 18$, $P < 0.001$). Among extensive metabolisers, all studies observed that metabolic ratios below 1.0, likely to reflect the homozygous dominant phenotype, were more common in lung cancer cases than in controls. The increased risk in extensive metabolisers appears to apply particularly to squamous cell and small cell cancer but is not statistically significant for adenocarcinoma (Caporaso *et al.*, 1989).

### Preliminary studies of phase II conjugation

Cancer risk is likely to be increased by *low* phase II conjugating enzyme activity, since phase I intermediate compounds if slowly conjugated would appear more likely to form DNA adducts. Thus slow acetylators (homozygous recessives) are at increased risk of occupational bladder cancer (Cartwright *et al.*, 1982). Acetylation is not associated with lung cancer risk (Philip *et al.*, 1988; Roots *et al.*, 1988), but conjugating enzymes involved in PAH metabolism (Figure 1) may be. Oesch *et al.* (1980) found little difference between cases and controls in conjugating enzyme activity in lung tissue, but Seidegard *et al.* (1986), using peripheral blood monocytes, found that high glutathione transferase activity was less common in lung cancer cases than controls. Among heavy smokers, high activity was found in 14 out of 46 cases (30%) and 38 out of 65 controls (59%) ($P < 0.01$). This result is preliminary, has not yet been confirmed or refuted by others, and is prone to the various biases discussed above. Smoking for example reduces glutathione transferase activity, so that the matching of cases and controls in recent smoking habit is critical.

Glutathione transferase activity may be dominated by a single autosomal gene. In a study of 248 individuals (Seidegard & Pero, 1985) activity was trimodal. Comparison with Hardy–Weinberg expected values, and a family study, suggested that very high (8% of the population), high (38%) and low (54%) activity represented homozygous dominant, heterozygous dominant and homozygous recessive genotypes respectively.

### DNA sequences as markers of lung cancer risk

Future developments in elucidating metabolic predisposition to cancer are likely to be dominated by developments in recombinant DNA technology. There is much recent activity in this area: 67 complete P-450 complementary DNA or protein sequences were known in 1987 (Nebert & Gonzalez, 1987). Jaiswal *et al.* (1985) have sequenced the human $P_1$-450 gene and shown that the quantity of $P_1$m-RNA correlates with induced AHH activity in human lymphocytes, but no correlation of the $P_1$-450 gene with risk of any cancer has yet been published. Jaiswal *et al.* (1987) have also reported the complementary DNA and protein sequences for $P_3$-450. The gene responsible for debrisoquine hydroxylation has been identified as a structural gene, its complementary DNA has been cloned and expressed in cell culture, and three different mutant genes have been shown to give rise to defective messenger-RNA splicing, resulting in the absence of an immunodetectable protein in the liver (Gonzalez *et al.*, 1988; Skoda *et al.*, 1988). The three mutant genes so far identified, however, account for only about half of all poor metabolisers.

This approach has the potential to detect single genes that predispose to lung cancer, identifying genotype rather than phenotype, and avoiding the biases involved in the metabolic studies. It has as yet no advantage over the pharmacological measure of debrisoquine hydroxylation, because it cannot distinguish all poor metabolisers from extensive metabolisers. Also, no probe for the conjugating enzymes is imminent. However, 'screening' of newly sequenced P-450 genes for association with lung cancer could greatly facilitate the search for genetic determinants of lung cancer risk.

**Table V** Debrisoquine metaboliser phenotype in lung cancer cases and controls in four studies (EM = extensive metaboliser, PM = poor metaboliser)

| | | No. of cases | No. of controls | Odds ratio* (95% confidence limits) |
|---|---|---|---|---|
| Ayesh *et al.* | EM | 241 | 213 | 5.9 (2.0, 18) |
| (1984) | PM | 4 | 21 | |
| Roots *et al.* | EM | 251 | 240 | 1.7 (0.9, 3.0) |
| (1988) | PM | 19 | 30 | |
| Caporoso *et al.* | EM | 62 | 54 | 4.0 (0.8, 20) |
| (1988) | PM | 2 | 7 | |
| Law *et al.* | EM | 102 | 95 | 4.8 (1.0, 23) |
| (1989) | PM | 2 | 9 | |

*Estimating relative risk of lung cancer in EM compared to PM.

### Public health application

Evidence for genetic predisposition to lung cancer is sought primarily to increase our understanding of the biology of cancer. Future public health application is unlikely. It would be difficult to screen cigarette smokers for lung cancer risk without at the same time implying that those without the marker could smoke safely. Lung cancer accounts for only about a third of all deaths attributable to smoking, and there is no reason to suppose that the risk of coronary heart disease and other smoking-related diseases would be different in subjects with a marker for lung cancer risk. The harm of such screening could outweigh the benefit.

A more limited application could lie in the selection of low-risk workers for occupations involving risk of lung cancer (Caporaso *et al.*, 1989). Even this application must be improbable. A moderate or high concentration of carcinogens in an industrial environment could be justified only by the identification of a minority of the population having a lung cancer risk of virtually zero. It would not for example be acceptable to submit poor metabolisers of debrisoquine (having about one-quarter the lung cancer risk of extensive metabolisers) to such an exposure.

### Conclusions

Genetic factors must contribute to lung cancer risk, since it is the metabolites of environmental carcinogens that usually initiate a cancer, and the metabolic pathways are genetically controlled. The studies reviewed have examined the question of whether significant genetically determined variation in risk exists between individuals, and some of the evidence suggests that it does.

The studies of familial clustering are too insensitive to be useful, and are consistent with any interpretation from a substantial genetic effect to no genetic effect at all; a marker is needed to demonstrate genetic predisposition. Associations of lung cancer with two naturally occurring antigens, the a4 Aa-*ras* allele and the HLA-B12 allele, may be important but they require confirmation. The evidence linking small cell lung cancer with the retinoblastoma (Rb) gene and with 3p chromosomal deletions is suggestive, and there is good evidence for genetic predisposition in at least some cases of the rare alveolar cell carcinoma, which is not smoking-related.

The numerous studies of PAH metabolism in smoking-related lung cancer cannot collectively constitute convincing evidence for genetic predisposition to lung cancer. *Ah* dominant mice showed predisposition to PAH-induced lung cancer, but this experimental model need not be relevant to tobacco smoking in humans. The control of AHH activity in humans is polygenic. The case–control studies measuring AHH activity directly in lung tissue, one lymphocyte study in which the cancer in cases had been resected (reducing possible bias from biological effects of cancer), and the two lymphocyte studies that measured DNA binding were all negative (Tables II and III). One study has shown higher induced AHH activity in lung cancer, but non-carcinogenic metabolites were measured remote from the lung, and some sources of bias could not be excluded. The one positive study cannot outweigh the negative evidence. The association of low conjugating enzyme activity with lung cancer in one study is interesting, but the result may have been produced by differences in smoking habit or other bias and requires confirmation.

Antipyrine is not a suitable drug to investigate the genetic determinants of lung cancer. Debrisoquine is, since 4-hydroxylation is its only major metabolic pathway and is dominated by a single gene. The greater risk of lung cancer in extensive compared to poor metabolisers of debrisoquine, confirmed in four studies, is compelling evidence for genetic predisposition. No known environmental factor could plausibly account for this difference, and it is in any case uncommon for environmental factors to have so powerful an effect on mono-oxidation as to reproduce the 10–200-fold genetically determined difference in mean metabolic ratio between poor and extensive debrisoquine metabolisers. Debrisoquine 4-hydroxylation is not associated with the mono-oxygenation of either PAHs or nitosamines, but there could be a shared metabolic pathway with another carcinogenic tobacco smoke constituent. Alternatively, however, the association with lung cancer might be explained by linkage disequilibrium, the gene regulating debrisoquine hydroxylation being chromosomally linked to another gene that directly affects lung cancer risk. There is thus no definite evidence for a metabolic mechanism in genetic predisposition to lung cancer. An ongogene for example might account for the associations of lung cancer with both debrisoquine metabolism and the retinoblastoma gene.

The size of the genetically determined variation between individuals in lung cancer risk is unresolved. To propose a genetic basis for the observation that only about 15% of smokers develop lung cancer implies that a minority of the population is at high risk, but there is little evidence for this. The debrisoquine studies identify homozygous recessives, 10% of the population, at about a 4-fold *reduced* risk; homozygous dominants are likely to be at higher risk than heterozygous dominants, if the two could be reliably distinguished, but the difference in risk would not be large. The studies of naturally occurring antigens suggest that about 15–25% of the population have about a 2-fold increased risk (though an incomplete association with the antigen that serves as a marker could dilute the real risk), and the unconfirmed conjugating enzyme study suggests a 2.5–3-fold increased risk in about half the population. In summary, there is some variation between individuals in genetically determined lung cancer risk, but as yet no evidence for substantially increased risk in a minority of the population.

The main interest of this work is the understanding of the biology of cancer, not public health. To screen smokers for genetically determined lung cancer risk is impractical because so many other diseases are also caused by smoking. An occupational application might be feasible but this is unlikely and certainly not imminent.

Any future metabolic studies should avoid the various biases (mostly consequent on the illness of the cases) that have affected the existing studies. This could be done in a longitudinal study, metabolic activity being measured (or blood or urine stored) in healthy smokers and a comparison made between those who subsequently developed lung cancer and matched controls who did not. Trell *et al.* (1985) have commenced such a study measuring AHH activity. Many subjects and long follow-up would be necessary. Alternatively, the bias inherent in previous case–control studies might be minimised by studying only long-term (say 5-year) survivors of resection for lung cancer. Bias associated with drugs, hospital stay and illness would be eliminated, and bias from a metabolic effect of cancer substantially avoided. This type of study could be performed quickly and economically. However, developments in molecular genetics promise attractive studies that would circumvent all bias. This approach is likely to dominate future research.

I thank Professor N. Wald (Chairman), Professor M. Bobrow, Dr R. Porter and other members of the MRC Smoking Research Review Committee, and Professor W. Bodmer, Dr J. Idle, Dr R. Meehan and Dr C.R. Wolf for assistance and for commenting on the manuscript.

### References

AIRD, L., BENTALL, H.H., MEHIGAN, A.A. & FRASER-ROBERTS, J.A. (1954). The blood groups in relation to peptic ulceration and carcinoma of colon, rectum, breast and bronchus. *Br. Med. J.*, **ii**, 315.

AMBRE, J., GRAEFF, D., BURES, F., HAUPT, D. & DEASON, K. (1977). Antipyrine metabolism and bronchogenic carcinoma. *J. Med.*, **8**, 57.

AMSBAUGH, S.C., DING, J.-H., SWAN, D.C., POPESCU, N.C. & CHEN, Y.-T. (1986). Expression and chromosomal localization of the cytochrome $P_1$-450 gene in human mitogen-stimulated lymphocytes. *Cancer Res.*, **46**, 2423.

ARNOTT, M.S., YAMAUCHI, T. & JOHNSTON, D.A. (1979). Aryl hydrocarbon hydroxylase in normal and cancer populations. In *Carcinogens: Identification and Mechanisms of Action*, Griffin, A.C. & Shaw, R.C. (eds) p. 145. Raven Press: New York.

ASHLEY, D.J.B. (1969). Blood groups and lung cancer. *J. Med. Genet.*, **6**, 183.

ATLAS, S.A., VESELL, E.S. & NEBERT, D.W. (1976). Genetic control of inter-individual variations in the inducibility of aryl hydrocarbon hydroxylase in cultured human lymphocytes. *Cancer Res.*, **36**, 4619.

AYESH, R. & IDLE, J.R. (1985). Evaluation of drug oxidation phenotypes in the biochemical epidemiology of lung cancer risk. In *Microsomes and Drug Oxidation*, Boobis, A.R., Caldwell, J., De Matteis, F. & Elcombe, C.R. (eds) p. 340. Taylor and Francis: London.

AYESH, R., IDLE, J.R., RITCHIE, J.C., CROTHERS, M.J. & HETZEL, M.R. (1984). Metabolic oxidation phenotypes as markers for susceptibility to lung cancer. *Nature*, **312**, 169.

BECKMAN, G., EKLUND, A., FROHLANDER, N. & STJERNBERG, N. (1986). Haptoglobin groups and lung cancer. *Hum. Hered.*, **36**, 258.

BIRRER, M.J. & MINNA, J.D. (1988). Molecular genetics of lung cancer. *Semin. Oncol.*, **15**, 226.

BLAIN, P.G., MUCKLOW, J.C., WOOD, P., ROBERTS, D.F. & RAWLINS, M.D. (1982). Family study of antipyrine clearance. *Br. Med. J.*, **284**, 150.

BODMER, W.F. (1986). Genetic susceptibility to cancer. In *Accomplishments in Cancer Research*, Fortner, J.G. & Rhoads, J.E. (eds) p. 198. J.B. Lippincott: Philadelphia.

BOOBIS, A.R., BRODIE, M.J., KAHN, G.C. & 4 others (1981). Comparison of the *in vivo* and *in vitro* rates of formation of the three main oxidative metabolites of antipyrine in man. *Br. J. Clin. Pharmacol.*, **12**, 771.

BRANCH, R.A., HERBERT, C.M. & READ, A.E. (1973). Determinants of serum antipyrine half lives in patients with liver disease. *Gut*, **14**, 569.

CANTRELL, E., BUSBEE, D., WARR, G. & MARTIN, R. (1973). Induction of aryl hydrocarbon hydroxylase in human lymphocytes and pulmonary alveolar macrophages – a comparison. *Life Sci.*, **13**, 1649.

CAPORASO, N., HAYES, R., DOSEMECI, M. & 4 others (1989). Lung cancer risk, occupational exposure and the debrisoquine metabolic phenotype. *Cancer Res.*, **49**, 3675.

CAPORASO, N., HOOVER, R., AISNER, S. & 5 others (1988). Debrisoquine metabolic phenotype and the risk of lung cancer. *Proc. Am. Soc. Clin. Oncol.*, **8**, 336.

CARTWRIGHT, R.A., GLASHAN, R.W., ROGERS, H.J. & 4 others (1982). Role of *N*-acetyltransferase phenotypes in bladder carcinogenesis: a pharmacogenetic epidemiological approach to bladder cancer. *Lancet*, **ii**, 842.

COBB, R.R., STOMING, T.A. & WHITNEY, J.B. (1987). The aryl hydrocarbon hydroxylase (*Ah*) and a novel restriction fragment length polymorphism (RFLP) are located on mouse chromosome 12. *Biochem. Genet.*, **25**, 401.

COHEN, G.M., MEHTA, R. & MEREDITH-BROWN, M. (1979). Large interindividual variations in metabolism of benzo[a]pyrene by peripheral lung tissue from lung cancer patients. *Int. J. Cancer*, **24**, 129.

CONNEY, A.H. (1982). Induction of microsomal enzymes by foreign chemicals and carcinogenesis by polycyclic aromatic hydrocarbons: GHA Clowes Memorial Lecture. *Cancer Res.*, **42**, 4875.

CUMMING, J.F. (1976). The effect of arterial oxygen tension on antipyrine half-time in plasma. *Clin. Pharmacol. Ther.*, **19**, 468.

DANHOF, M. (1980). Antipyrine metabolite profile as a tool in the assessment of the activity of different drug oxidising enzymes in man. Thesis, University of Leiden, The Netherlands.

DANHOF, M. & BREIMER, D.D. (1979). Studies on the different metabolic pathways of antipyrine in man I: oral administration of 250, 500 and 1000 mg to healthy volunteers. *Br. J. Clin. Pharmacol.*, **8**, 529.

EICHELBAUM, M. (1984). Polymorphic drug oxidation in humans. *Fedn Proc.*, **43**, 2298.

EICHELBAUM, M., BAUR, M.P., DENGLER, H.J. & 4 others (1987). Chromosomal assignment of human chromosome P-450 (debrisoquine/sparteine type) to chromosome 22. *Br. J. Clin. Pharmacol.*, **23**, 455.

EMERY, A.E.H., ANAND, R., DANFORD, N., DUNCAN, W. & PATON, L. (1978). Aryl hydrocarbon hydroxylase inducibility in patients with cancer. *Lancet*, **i**, 470.

EVANS, D.A.P., MAHGOUB, A., SLOAN, T.P., IDLE, J.R. & SMITH, R.L. (1980). A family and population study of the genetic polymorphism of debrisoquine oxidation in a white British population. *J. Med. Genet.*, **17**, 102.

FARBER, E. (1981). Chemical carcinogenesis. *N. Engl. J. Med.*, **305**, 1379.

FORD, C.H.J., NEWMAN, C.E. & MACKINTOSH, P. (1981). HLA frequency and prognosis in lung cancer. *Br. J. Cancer*, **43**, 610.

GAHMBERG, L.G., SEKKI, A., KOSUNEN, T.U., HOLSTI, L.R. & MAKELA, O. (1979). Induction of aryl hydrocarbon hydroxylase activity in pulmonary carcinoma. *Int. J. Cancer*, **23**, 302.

GELBOIN, H.V. (1980). Benzo[a]pyrene metabolism, activation and carcinogenesis: role and regulation of mixed function oxidases and related enzymes. *Physiol. Rev.*, **60**, 1107.

GONZALEZ, F.J., JAISWAL, A.K. & NEBERT, D.W. (1986). P450 genes: evolution, regulation, and relationship to human cancer and pharmacogenetics. *Cold Spring Harbor Symp. Quant. Biol.*, **51**, 879.

GONZALEZ, F.J., SKODA, R.C., KIMURA, S. & 6 others (1988). Deficient metabolism of debrisoquine and other drugs is due to defective allele of a human P450 gene. *Nature*, **331**, 442.

GUIRGIS, H.A, LYNCH, H.T., MATE, T. & 6 others (1976). Aryl hydrocarbon hydroxylase activity in lymphocytes from lung cancer patients and normal controls. *Oncology*, **33**, 105.

GURTOO, H.L., MINOWADA, J., PAIGEN, B., PARKER, N.B. & HAYNER, N.T. (1977). Factors influencing the measurement and the reproducibility of aryl hydrocarbon hydroxylase activity in cultured human lymphocytes. *J. Natl Cancer Inst.*, **59**, 787.

HANKINSON, O., ANDERSON, R.D., BIRREN, B.W., SANDER, F., NEGLISHI, M. & NEBERT, D.W. (1985). Mutations affecting the regulation of transcription of the cytochrome $P_1$-450 gene in the mouse hepa-1 cell line. *J. Biol. Chem.*, **260**, 1790.

HARBOUR, J.W., LAI, S.-L., WHANG-PENG, J., GAZDAR, A.F., MINNA, J.D. & KAYE, F.J. (1988). Abnormalities in structure and expression of the human retinoblastoma gene in SCLC. *Science*, **241**, 353.

HARRIS, C.C., AUTRUP, H., CONNOR, R., BARRETT, L.A., MCDOWELL, E.M. & TRUMP, B.F. (1976). Interindividual variation in binding of benzo[a]pyrene to DNA in cultured human bronchi. *Science*, **194**, 1067.

HARRIS, C.C., AUTRUP, H., STONER, G. & 9 others (1977). Metabolism of benzo[a]pyrene and 7, N12-dimethylbenz[a]anthracene in cultured human bronchus and pancreatic duct. *Cancer Res.*, **37**, 3349.

HARRIS, C.C., MULVIHILL, J.J., THORGEIRSSON, S.S. & MINNA, J.D. (1980). Individual differences in cancer susceptibility. *Ann. Intern. Med.*, **92**, 809.

HART, P., FARRELL, G.C., COOKSLEY, W.G.E. & POWELL, L.W. (1976). Enhanced drug metabolism in cigarette smokers. *Br. Med. J.*, **ii**, 147.

HEIGHWAY, J., THATCHER, N., CERNY, T. & HASLETON, P.S. (1986). Genetic predisposition to human lung cancer. *Br. J. Cancer*, **53**, 453.

HILDEBRAND, C.E., GONZALEZ, F.J., KOZAK, C.M. & NEBERT, D.W. (1985). Regional linkage analysis of the dioxin inducible P-450 gene family on mouse chromosome 9. *Biochem. Biophys. Res. Commun.*, **130**, 396.

HUTTER, R.V.P. (1988). Cancer prevention and detection: status report and future prospects. *Cancer*, **61** (suppl.), 2372.

IDLE, J.R., MAHGOUB, A., ANGELO, M.M., DRING, L.G., LANCASTER, R. & SMITH, R.L. (1979). The metabolism of [$^{14}$C]-debrisoquine in man. *Br. J. Clin. Pharmacol.*, **7**, 257.

ISRAEL, D.I. & WHITLOCK, J.P. (1984). Regulation of sytochrome p1-450 gene transcription by 2,3,7,8-tetrachlorodibenzo-p-dioxin in wild type and varient mount heptoma cells. *J. Biol. Chem.*, **259**, 5400.

JAISWAL, A.K., GONZALEZ, F.J. & NEBERT, D.W. (1985). Human $P_1$-450 sequence and correlation of mRNA with genetic differences in benzo[a]pyrene metabolism. *Nucleic Acids Res.*, **13**, 4503.

JAISWAL, A.K., NEBERT, D.W., MCBRIDE, O.W. & GONZALEZ, F.J. (1987). Human $P_3$-450: cDNA and complete protein sequence, repetitive sequences in the 3′ nontranslated region, and localization of gene to chromosome 15. *J. Exp. Pathol.*, **3**, 1.

JAKOUBKOVA, J. & MAJSKY, A. (1965). Blood groups and neoplastic disease. *Neoplasma*, **12**, 611.

JETT, J.R., BRANUM, E.L., FONTANA, R.S., TAYLOR, W.F. & MOSES, H.L. (1979). Macromolecular binding of $^3$H-benzo[a]pyrene metabolites and lymphocyte transformation in patients with lung cancer, and in smoking and non-smoking control subjects. *Am. Rev. Respir. Dis.*, **120**, 369.

JETT, J.R., MOSES, H.L., BRANUM, E.L., TAYLOR, W.F. & FONTANA, R.S. (1978). Benzo[a]pyrene metabolism and blast transformation in peripheral blood mononuclear cells from smoking and non-smoking populations and lung cancer patients. *Cancer*, **41**, 192.

JOISHY, S. (1977). Alveolar cell carcinoma in twins, similarity in time of onset, histochemistry and site of metastasis. *Ann. Intern. Med.*, **87**, 447.

KALAMEGHAM, R., KRISHNASWAMY, K., KRISHNAMURTHY, S. & BHARGAVA, R.N.K. (1979). Metabolism of drugs and carcinogens in man: antipyrin elimination as an indicator. *Clin. Pharmacol. Ther.*, **25**, 67.

KAPITULNIC, J., POPPERS, P.J. & CONNEY, A.H. (1977). Comparative metabolism of benzo[a]pyrene and drugs in human liver. *Clin. Pharmacol. Ther.*, **21**, 166.

KARKI, N.T. & HUHTI, E. (1978). Aryl hydrocarbon hydroxylase activity in cultured lymphocytes from lung carcinoma patients and cigarette smokers. In *Abstracts of the Seventh International Congress of Pharmacology*, p. 254. Pergamon Press: Paris.

KARKI, N.T., POKELA, R., NUUTINEN, L. & PELKONEN, O. (1987). Aryl hydrocarbon hydroxylase in lymphocytes and lung tissue from lung cancer patients and controls. *Int. J. Cancer*, **39**, 565.

KATO, R., TAKANAKA, A., TAKAHASHI, A. & 4 others (1968). Drug metabolism enzymes in tumor-bearing rats. *Jpn. J. Pharmacol.*, **18**, 224.

KELLERMANN, G., JETT, J.R., LUYTEN-KELLERMANN, M., MOSES, H.L. & FONTANA, R.S. (1980). Variation of microsomal mixed function oxidase(s) and human lung cancer. *Cancer*, **45**, 1438.

KELLERMANN, G., LUYTEN-KELLERMANN, M., & SHAW, C.R. (1973*a*). Genetic variation of aryl hydrocarbon hydroxylasse in human lymphocytes. *Am. J. Hum. Genet.*, **25**, 237.

KELLERMANN, G., SHAW, C.R. & LUYTEN-KELLERMANN, M. (1973*b*). Aryl hydrocarbon hydroxylase inducibility and bronchogenic carcinoma. *N. Engl. J. Med.*, **289**, 934.

KORSGAARD, R., STIKSA, G., SIMONSSON, B.G. & TRELL, E. (1977). Smoking habits and aryl hydrocarbon hydroxylase inducibility in patients with malignant tumours of the respiratory tract. *Scand. J. Respir. Dis.*, Suppl. **99**, 50.

KORSGAARD, R. & TRELL, E. (1978). Aryl hydrocarbon hydroxylase and bronchogenic carcinomas associated with smoking. *Lancet*, **i**, 1103.

KOURI, R.E., BILLUPS, L.H., RUDE, T.H., WHITMIRE, C.E., SASS, B. & HENRY, C.J. (1980). Correlation of inducibility of aryl hydrocarbon hydroxylase with susceptibility to 3-methylcholanthrene-induced lung cancers. *Cancer Lett.*, **9**, 277.

KOURI, R.E., MCKINNEY, C.E., SLOMIANY, D.R., SNODGRASS, N.P., WRAY, N.P. & MCLEMORE, T.L. (1982). Positive correlation between high aryl hydrocarbon hydroxylase activity and primary lung cancer as analysed in cryopreserved lymphocytes. *Cancer Res.*, **42**, 5030.

LANG, M.A. & NEBERT, D.W. (1981). Structural gene products of the *Ah* locus. Evidence for many unique P-450-mediated mono-ozygenase activities reconstituted from 3-methylcholanthrene-treated C57BL/6N mouse liver microsomes. *J. Biol. Chem.*, **256**, 12058.

LAW, M.R., HETZEL, M.R. & IDLE, J.R. (1989). Debrisoquine metabolism and genetic predisposition to lung cancer. *Br. J. Cancer*, **59**, 686.

LEONARD, R.C.F., MACKAY, T., BROWN, A., GREGOR, A., CROMPTON, G.K. & SMYTH, J.F. (1988). Small-cell lung cancer after retinoblastoma. *Lancet*, **ii**, 1503.

LIEBERMAN, J. (1978). Aryl hydrocarbon hydroxylase in bronchogenic carcinoma. *N. Engl. J. Med.*, **298**, 686.

LYNCH, H.T., FAIN, P.R., ALBANO, W.A. & 4 others (1982). Genetic/epidemiological findings in a study of smoking associated tumours. *Cancer Genet. Cytogenet.*, **6**, 163.

LYNCH, H.T., KIMBERLING, W.J., MARKVICKA, S.E. & 4 others (1986). Genetics and smoking-associated cancers. *Cancer*, **57**, 1640.

MCCONNELL, R.B., CLARKE, C.A. & DOWNTON, F. (1955). Blood groups in carcinoma of the lung. *Br. Med. J.*, **ii**, 674.

MCLEMORE, T.L., MARTIN, R.R. & BUSBEE, D.L. (1977). Aryl hydrocarbon hydroxylase activity in pulmonary macrophages and lymphocytes from lung cancer and non-cancer patients. *Cancer Res.*, **37**, 1175.

MCLEMORE, T.L., MARTIN, R.R., PICKARD, L.R. & 7 others (1978*a*). Analysis of aryl hydrocarbon hydroxylase activity in human lung tissue, pulmonary macrophages, and blood lymphocytes. *Cancer*, **41**, 2292.

MCLEMORE, T.L., MARTIN, R.R., WRAY, N.P., CANTRELL, E.T. & BUSBEE, D.L. (1978*b*). Dissociation between aryl hydrocarbon hydroxylase activity in cultured pulmonary macrophages and blood lymphocytes from lung cancer patients. *Cancer Res.*, **38**, 3805.

MCLEMORE, T.L., MARTIN, R.R., SPRINGER, R.R., WRAY, N., CANTRELL, E.T. & BUSBEE, D.L. (1979). Aryl hydrocarbon hydroxylase activity in pulmonary alveolar macrophages and lymphocytes from lung cancer and non-cancer patients: a correlation with family histories of cancer. *Biochem. Genet.*, **17**, 795.

MARKMAN, M., BRAINE, H.G. & ABELOFF, M.D. (1984). Histocompatability antigens in small cell carcinoma of the lung. *Cancer*, **54**, 2943.

MEEHAN, R.R., GOSDEN, J.R., ROUT, D. & 9 others (1988*a*). Human cytochrome P-450 PB-1: a multigene family involved in mephenytoin and steroid oxidations that maps to chromosome 10. *Am. J. Hum. Genet.*, **42**, 26.

MEEHAN, R.R., SPEED, R.M., GOSDEN, J.R. & 10 others (1988*b*). Chromosomal organisation of the cytochrome P-450-2C gene family in the mouse: a locus associated with constitutive aryl hydrocarbon hydroxylase. *Proc. Natl Acad. Sci. USA*, **85**, 2662.

MILLER, E.C. (1978). Some current perspectives on chemical carcinogenesis in humans and experimental animals: Presidential address. *Cancer Res.*, **38**, 1479.

MILLER, J.A. & MILLER, E.C. (1983). The metabolic activation and nucleic acid adducts of naturally occurring carcinogens: recent results with ethyl carbamate and the spice flavors safrole and estragole. *Br. J. Cancer*, **48**, 1.

NEBERT, D.W. (1981). Possible clinical importance of genetic differences in drug metabolism. *Br. Med. J.*, **283**, 537.

NEBERT, D.W., ADESNIK, M., COON, M.J. & 10 others (1987). The P-450 gene super family: recommended nomenclature. *DNA*, **6**, 1.

NEBERT, D.W. & GONZALEZ, F.J. (1987). P450 genes: structure, evolution, and regulation. *Ann. Rev. Biochem.*, **56**, 945.

NEBERT, D.W., GOUJON, F.M. & GIELEN, J.E. (1972). Aryl hydrocarbon hydroxylase induction by polycyclic hydrocarbons: simple autosomal dominant trait in the mouse. *Nature*, **236**, 107.

NEGISHI, M. & NEBERT, D.W. (1979). Structural gene products of the *Ah* locus. Genetic and immunochemical evidence for two forms of mouse liver cytochrome P-450 induced by 3-methylcholanthrene. *J. Biol. Chem.*, **254**, 11015.

OCRAFT, K.P., MUSKETT, J.M. & BROWN, S. (1985). Localisation of the aryl hydrocarbon hydroxylase gene to the 2q31-2pter of chromosome 2. *Ann. Hum. Genet.*, **49**, 237.

OESCH, F., SCHMASSMANN, H., OHNHAUS, E., ALTHAUS, U. & LORENZ, J. (1980). Mono-oxygenase, epoxide hydrolase, and glutathione-S-transferase activities in human lung. Variation between groups of bronchogenic carcinoma and non-cancer patients and interindividual differences. *Carcinogenesis*, **1**, 827.

OKEY, A.B., BONDY, G.P., MASON, M.E. & 4 others (1980). Temperature-dependent cytosol-to-nucleus translocation of the Ah receptor for 2,3,7,8-tetrachlorodibenzo-p-dioxin in continuous cell culture lines. *J. Biol. Chem.*, **253**, 11415.

OKUDA, T., VESELL, E.S., PLOTKIN, E., TARONE, R., BAST, R.C. & GELBOIN, H.V. (1977). Interindividual and intraindividual variations in aryl hydrocarbon hydroxylase in monocytes from monozygotic and dizygotic twins. *Cancer Res.*, **37**, 3904.

O'MALLEY, K., CROOKES, J., DUKE, E. & STEVENSON, I.H. (1971). Effect of age and sex on human drug metabolism. *Br. Med. J.*, **iii**, 607.

OOI, W.L., ELSTON, R.C., CHEN, V.W., BAILEY-WILSON, J.E. & ROTHSCHILD, H. (1986). Increased familial risk for lung cancer. *J. Natl Cancer Inst.*, **76**, 217.

PAIGEN, B., WARD, E., STEENLAND, K., HOUTEN, L., GURTOO, H.L. & MINOWADA, J. (1978). Aryl hydrocarbon hydroxylase in cultured lymphocytes of twins. *Am. J. Hum. Genet.*, **30**, 561.

PAIGEN, B., GURTOO, H.L., MINOWADA, J. & 4 others (1977). Questionable relation of aryl hydrocarbon hydroxylase to lung cancer risk. *N. Engl. J. Med.*, **297**, 346.

PARKER, G. & WALSH, R.J. (1958). Blood groups and disease: Carcinoma of various organs. *Med. J. Aust.*, **ii**, 835.

PAUL, S.M., BACHARACH, B. & GOEPP, C. (1987). A genetic influence on alveolar cell carcinoma. *J. Surg. Oncol.*, **36**, 249.

PELKONEN, O. & NEBERT, D.W. (1982). Metabolism of polycyclic aromatic hydrocarbons: etiologic role in carcinogenesis. *Pharmacol. Rev.*, **34**, 189.

PETO, J. (1980). Genetic predisposition to cancer. Banbury Report 4: Cancer Incidence in Defined Populations. Cold Spring Harbour Laboratory, p.203.

PHILIP, P.A., FITZGERALD, D.L., CARTWRIGHT, R.A., PEAKE, M.D. & ROGERS, H.J. (1988). Polymorphic *N*-acetylation capacity in lung cancer. *Carcinogenesis*, **9**, 491.

RENNIE, H.M. & HABER, R.W. (1961). Blood groups in carcinoma of the lung. *Med. J. Aust.*, **ii**, 61.

ROBERTS, T.E., HASTLETON, P., SWINDELL, R. & LAWSON, R. (1988). Blood groups and lung cancer. *Br. J. Cancer*, **58**, 278.

ROOTS, I., DRAKOULIS, N., PLOCH, M. & 6 others (1988). Debrisoquine hydroxylation phenotype, acetylation phenotype and ABO blood groups as genetic host factors of lung cancer risk. *Klin. Wochenschr.*, **66** (suppl. XI), 87.

ROSSO, R., DONELLI, M.G., FRANCHI, G. & GARATTINI, S. (1971). Impairment of drug metabolism in tumor-bearing animals. *Eur. J. Cancer*, **7**, 565.

RUDIGER, H.W., HEISIG, V. & HAIN, E. (1980). Enhanced benzo[a]pyrene metabolism and formation of DNA adducts in monocytes of patients with lung cancer. *J. Cancer Res. Clin. Oncol.*, **96**, 295.

SABADIE, N., RICHTER-REICHHELM, H.B., SARACCI, R., MOHR, U. & BARTSCH, H. (1981). Interindividual differences in oxidative benzo[a]pyrene metabolism by normal and tumorous surgical lung specimens from 105 lung cancer patients. *Int. J. Cancer*, **27**, 417.

SEIDEGARD, J. & PERO, R.W. (1985). The hereditary transmission of high glutathione transferase activity towards trans-stilbene oxide in human mono-nuclear leucocytes. *Hum. Genet.*, **69**, 66.

SEIDEGARD, J., PERO, R.W., MILLER, D.G. & BEATTIE, E.J. (1986). A glutathione transferase in human leukocytes as a marker for the susceptibility to lung cancer. *Carcinogenesis*, **7**, 751.

SENGAR, D.P.S., MCLEISH, W.A., STUART, T.H.M. & HARRIS, J.E. (1977). HLA antigens in bronchogenic carcinoma. *Oncology*, **34**, 143.

SHIMADA, T., MISONO, K.S. & GUENGERICH, F.P. (1986). Human liver microsomal cytochrome P-450 mephenytoin 4-hydroxylase, a prototype of genetic polymorphism in oxidative drug metabolism. *J. Biol. Chem.*, **261**, 909.

SKODA, R.C., GONZALEZ, F.J., DEMIERRE, A. & MEYER, U.A. (1988). Two mutant alleles of the human cytochrome P-450dbl gene (P450C2D1) associated with genetically deficient metabolism of debrisoquine and other drugs. *Proc. Natl Acad. Sci. USA*, **85**, 5240.

SLOAN, T.P., LANCASTER, R., SHAH, R.R., IDLE, J.R. & SMITH, R.L. (1983). Genetically determined oxidation capacity and the disposition of debrisoquine. *Br. J. Clin. Pharmacol.*, **15**, 443.

SNODGRASS, D.R., MCLEMORE, T.L., TEAGUE, R.B., WRAY, N.P. & BUSBEE, D.L. (1981). Aryl hydrocarbon hydroxylase activity in pulmonary macrophages and blood lymphocytes. *Chest*, **80**, 42S.

STEINER, E., ISELIUS, L., ALVAN, G., LINDSTEN, J. & SJOQVIST, F. (1985). A family study of genetic and environmental factors determining polymorphic hydroxylation of debrisoquin. *Clin. Pharmacol. Ther.*, **38**, 394.

TAKASUGI, M., TERASAKI, P.I., HENDERSON, B., MICKEY, M.R., MENCK, H. & THOMPSON, R.W. (1973). HLA antigens in solid tumors. *Cancer Res.*, **33**, 648.

TOKUHATA, G.K. & LILLIENFELD, A.M. (1963*a*). Familial aggregation of lung cancer in humans. *J. Natl Cancer Inst.*, **30**, 289.

TOKUHATA, G.K. & LILLIENFELD, A.M. (1963*b*) Familial aggregation of lung cancer among hospital patients. *Public Health Rep.*, **78**, 277.

TONGIO, M.M., KERSCHEN, C., PAULI, G., ROESLIN, N., GRANGE, D. & MAYER, S. (1982). HLA antigens and primary bronchial carcinoma. *Cancer*, **49**, 2485.

TRELL, L., KORSGAARD, R., JANZON, L. & TRELL, E. (1985). Distribution and reproducibility of aryl hydrocarbon hydroxylase inducibility in a prospective population study of middle aged male smokers and non-smokers. *Cancer*, **56**, 1988.

TSCHANZ, C., HIGNITE, C.E., HUFFMAN, D.H. & AZARNOFF, D.L. (1977). Metabolic disposition of antipyrine in patients with lung cancer. *Cancer Res.*, **37**, 3881.

VESELL, E.S. (1979). The antipyrine test in clinical pharmacology: conceptions and misconceptions. *Clin. Pharmacol. Ther.*, **26**, 275.

VESELL, E.S., PAGE, J.G. & PASSANANTI, G.T. (1971). Genetic and environmental factors affecting ethanol metabolism in man. *Clin. Pharmacol. Ther.*, **12**, 192.

VESTAL, R.E., NORRIS, A.H., TOBIN, J.D., COHEN, B.H., SHOCK, N.W. & ANDRES, R. (1975). Antipyrine metabolism in man: influence of age, alcohol caffeine and smoking. *Clin. Pharmacol. Ther.*, **18**, 425.

WARD, E., PAIGEN, B., STEENLAND, K. & 4 others (1978). Aryl hydrocarbon hydroxylase in persons with lung or laryngeal cancer. *Int. J. Cancer*, **22**, 384.

WIEBEL, F.J., HLAVICA, P. & GRZESCHIK, K.H. (1981). Expression of aromatic polycyclic hydrocarbon-induced mono-oxygenase (aryl hydrocarbon hydroxylase) in man x mouse hydrids is associated with human chromosome 2. *Hum. Genet.*, **59**, 277.

WOLF, C.R. (1986). Cytochrome P-450s: polymorphic multigene families involved in carcinogen activation. *Trends Genet.*, **8**, 209.

WOLFF, T., DISTLERATH, L.M., WORTHINGTON, M.T. & 6 others (1985). Substrate specificity of human liver cytochrome P–450 debrisoquine 4-hydroxylase probed using immunochemical inhibition and chemical modeling. *Cancer Res.*, **45**, 2116.

YOKOTA, J., WADA, M., SHIMOSATO, Y., TERADA, M. & SUGIMURA, T. (1987). Loss of heterozygosity on chromosomes 3, 13 and 17 in small-cell carcinoma and on chromosome 3 in adenocarcinoma of the lung. *Proc. Natl Acad. Sci. USA*, **84**, 9252.